# Solar‐Driven Bifunctional Adsorption‐Storage Films for Ultra‐Fast Dehumidification and Freshwater Supply in Low‐Carbon Buildings

**DOI:** 10.1002/advs.76145

**Published:** 2026-06-18

**Authors:** Yuechao Chao, Junwei Liu, Zhihua Zhou, Yahui Du, Hailu Wei, Haibin Yang, Xueqing Yang, Cheng Wang, Zhenzhong Zeng, Hongzhi Cui, Jinyue Yan

**Affiliations:** ^1^ School of Environmental Science and Engineering Tianjin University Tianjin China; ^2^ International Centre of Urban Energy Nexus The Hong Kong Polytechnic University Hong Kong Kowloon China; ^3^ Department of Building Environment and Energy Engineering The Hong Kong Polytechnic University Hong Kong Kowloon China; ^4^ Key Laboratory or Resilient Infrastructures of Coastal Cities (MOE) College of Civil and Transportation Engineering Shenzhen University Shenzhen Guangdong China; ^5^ School of Environmental Science and Engineering Southern University of Science and Technology Shenzhen China

**Keywords:** atmospheric water harvesting, building energy saving, leak‐proof adsorption‐storage designs, solar‐driven regeneration, ultra‐fast dehumidification

## Abstract

Indoor humidity strongly affects both building energy demand and occupant health in hot and humid climates, but it remains largely controlled by energy‐intensive mechanical systems and is seldom exploited as a source of freshwater. Here, we demonstrate a passive humidity regulation and freshwater harvesting strategy enabled by cost‐effective adsorption films (18.79$·m^−2^) that couple moisture capture, storage, and release within indoor environments. The films rapidly reduce relative humidity from 90.7% to 21.6% within one hour while maintaining long‐term operational stability and release the captured moisture to produce freshwater at a rate of approximately 1.1 kg·m^−2^·day^−1^ under ambient conditions. Leveraging the harvested water, an autonomous plant irrigation system is achieved, enabling sustained regulation of indoor CO_2_ concentration (about 920.2 ppm) without manual intervention. Global‐scale projections indicate that this passive approach could reduce building energy consumption by up to 29.9 kWh·year^−1^·m^−2^ and associated carbon emissions by 16.5 kg·year^−1^·m^−2^, with an exceptionally short payback period of 48 days. This work reframes indoor humidity from a latent load to a recoverable resource, offering an integrated route towards water harvesting, energy reduction and healthier indoor environments in sustainable buildings.

## Graphical abstract

Bifunctional adsorption‐storage films enable autonomous indoor humidity regulation and freshwater harvesting, rapidly decreasing relative humidity from 90.7% to 21.6% within one hour while producing 1.1 kg·m^−2^·day^−1^ of water, offering a passive route towards energy‐efficient humidity control and sustainable indoor environments.

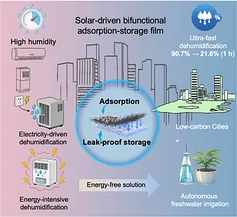

## 1 Introduction

Achieving the 1.5°C climate target under the Paris Agreement requires substantial reductions in carbon emissions, particularly in cities where energy demand is highly concentrated. Buildings constitute a major energy end‐use sector, and in subtropical metropolises such as Hong Kong, air‐conditioning systems may account for 30–50% of total building energy consumption, with latent‐load management contributing nearly half of this demand. Urban heat islands further intensify thermal stress, as dense infrastructure and limited vegetation elevate local temperatures, compared with surrounding rural regions. To maintain indoor comfort under persistently high temperature and humidity, conventional buildings rely heavily on mechanical cooling and dehumidification, which are energy‐intensive and contribute substantially to greenhouse gas emissions [1]. Excessive indoor humidity not only diminishes occupant comfort but also promotes the proliferation of pathogens and allergens and accelerates material degradation [2, 6]. In hot and humid urban environments, elevated humidity amplifies perceived heat stress, posing serious health risks to vulnerable populations [5, 8, 9]. Together, these energy, health and environmental pressures underscore the urgent need for passive, scalable approaches that can regulate indoor humidity while recovering freshwater without active energy input [3, 4, 18].

Current dehumidification technologies are dominated by vapour‐compression cooling and condensation processes embedded in air‐conditioning systems. However, the accumulation of condensate within these systems will promote bacterial growth, posing additional risks to indoor environmental quality [10, 11]. In recent years, passive adsorption‐based dehumidification strategies have attracted considerable attention as energy‐efficient alternatives to conventional air‐conditioning [12, 13]. Liquid and solid adsorbents, such as hygroscopic salts, silica gels, and zeolites, are widely explored for indoor humidity control [7]. Nevertheless, their practical implementation is limited by low adsorption capacity, high regeneration energy, and relatively short operational lifetimes. To overcome these challenges, metal‐organic frameworks (MOFs) [14, 15, 19, 20, 56], hygroscopic gels [21, 22, 23, 48], and salt‐based adsorbents [24, 25, 58] have been developed to enhance water harvesting efficiency. Despite their advantages, MOFs typically involve costly precursors, complex synthesis procedures, and stringent fabrication conditions [27, 28, 29]. Hygroscopic gels, while exhibiting excellent water retention, still suffer from rapid performance degradation and limited adsorption under low‐humidity conditions [16, 17]. Compared with MOFs and gels, low‐cost salt‐loaded porous adsorbents can capture moisture across a wide humidity range and release it via solar heating^62^, ^63^, ^64^, ^65^, ^66^, enabling efficient atmospheric water harvesting [16, 26]. Their facile fabrication and broad humidity compatibility highlight their strong commercial potential [30, 31]. However, most salt‐based adsorbents exhibit limited water storage capacity^51^, ^52^, ^53^, ^54^, ^57^, which restricts practical deployment due to leakage issues [32, 59, 60]. These challenges point to a critical gap in material design: the need for adsorbents that simultaneously provide high water uptake, robust storage, and facile desorption at low cost^45^, ^46^, ^47^.

**FIGURE 1:**
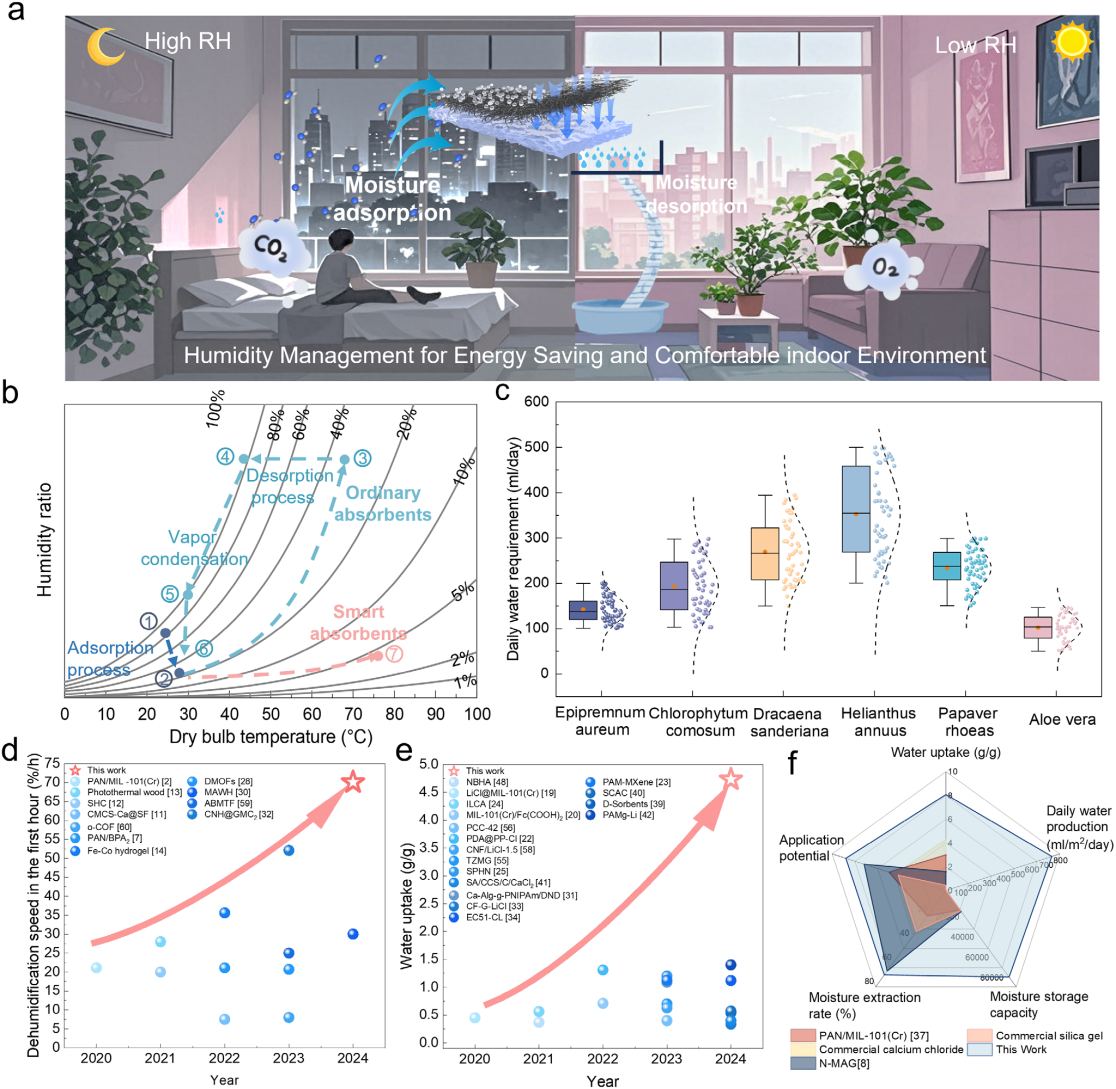
Schematic illustration of our humidity management strategy enabled by the bifunctional water adsorption–storage design. (a) Operating mechanism of the BASF for improving indoor air quality while simultaneously reducing building energy consumption. (b) Psychrometric representation of indoor dehumidification and water harvesting processes. For conventional hygroscopic materials, moisture adsorption proceeds from state 1 to state 2, followed by thermal desorption along the path 2‐3‐4. The released vapor subsequently condenses through states 4‐5‐6 to form liquid water. In contrast, smart hygroscopic materials undergo moisture uptake from state 1 to state 2 and, upon heating, and directly generate liquid water via a phase transition from state 2 to state 7, thereby bypassing the vapor‐phase condensation process. (c) Daily water consumption requirements of representative indoor plant species. (d) Progress of indoor dehumidification performance. (e) Progress of single cycle atmospheric water harvesting performance. (f) Comparative evaluation of the overall performance of our BASF relative to typical hygroscopic adsorbents.

Herein, we report a promising bifunctional adsorption‐storage film (BASF) that integrates porous nanofibers with adsorbent‐infused hydrogels. In this architecture, the adsorbent enables rapid moisture capture through strong hygroscopicity, the porous nanofibres facilitate solar‐driven desorption via photothermal conversion, and the hydrogel matrix provides leak‐proof water storage to ensure long‐term stability (Figure 1a). At night, the BASF rapidly adsorbs moisture, lowering relative humidity within one hour to maintain a comfortable indoor range. The stored water is retained securely, preventing bacterial and viral proliferation caused by leakage, thereby preserving indoor environmental health. During the daytime, our BASF harness solar heating to evaporate or release the captured water through smart hydrogel functionality (Figure 1b), achieving high water uptake and sustainable freshwater harvesting sufficiently to irrigate common indoor plants (Figure 1c). By supporting photosynthesis, these plants further help lower CO_2_ concentrations arising from human activities while increasing O_2_ levels, thereby improving indoor environmental quality. Compared with existing materials, our BASF demonstrate significantly higher dehumidification efficiency, superior water storage capacity, and enhanced freshwater production, while maintaining scalability and low cost (Figure 1d–f and Supplementary Table S1). Replacing conventional hydrogel with a smart one further enhances freshwater release, extending the application potential of our design (more details refer to *Experimental Methods*) [55]. Overall, this work provides a promising route toward energy‐free, cost‐effective indoor humidity management, simultaneously enabling freshwater harvesting and promoting healthy living environments.

## 2 Results and Discussions

### 2.1 Material Design and Structural Properties

**FIGURE 2:**
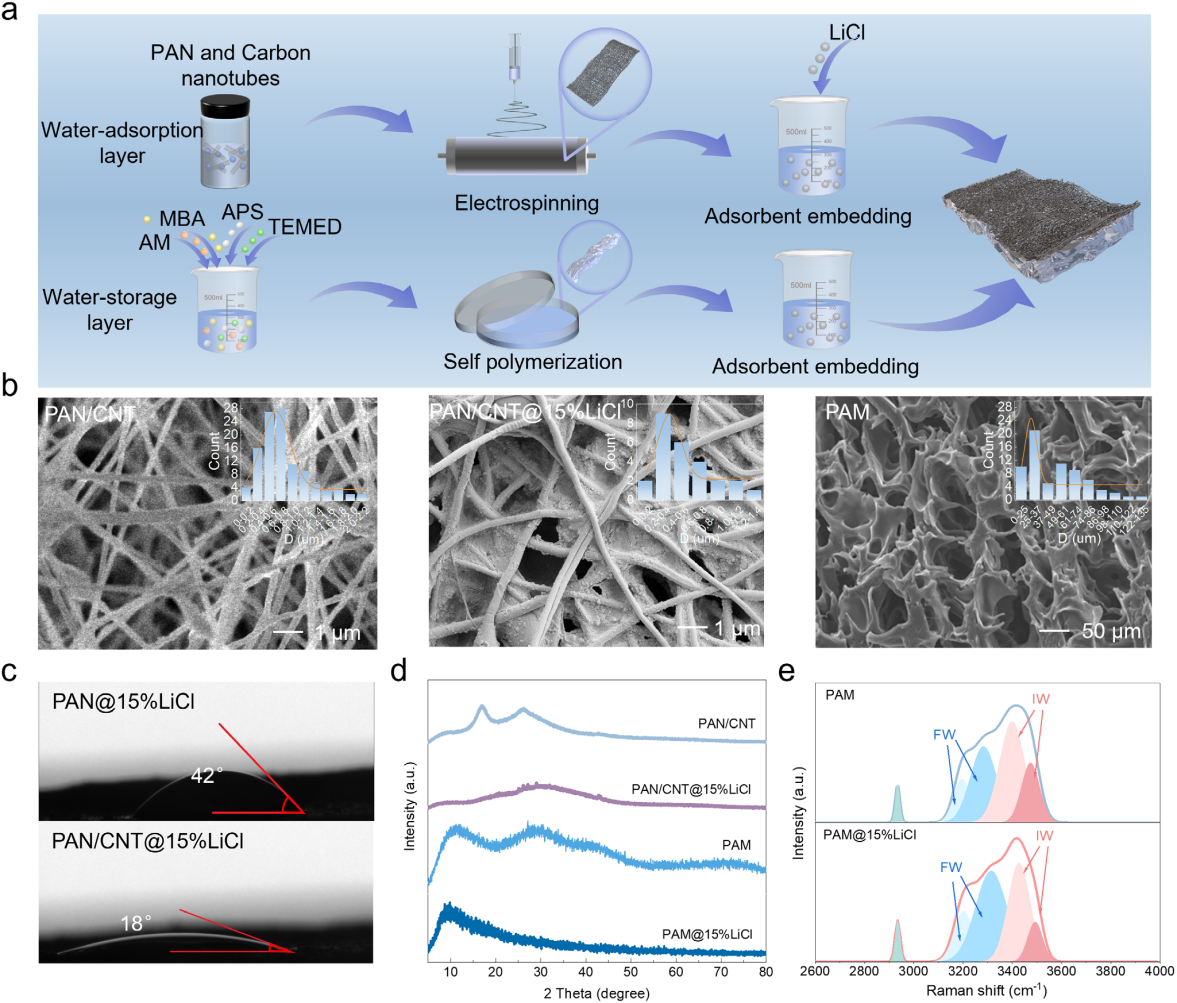
Design and fundamental characteristics of our BASF. (a) Schematic illustration of the fabrication procedure for the BASF. (b) Scanning Electron Microscopy (SEM) image of BASF. (c) Water contact angles of BASF. (d) X‐ray Diffraction (XRD) pattern of BASF. (e) Raman spectrum of BASF.

Developing adsorbent materials that combine strong water uptake, robust storage across a wide humidity range, and rapid regeneration under solar irradiation represents an effective strategy for indoor humidity management and freshwater supply [32, 33, 34]. Here, we designed a bifunctional composite adsorbent composed of hydrophilic polyacrylonitrile/carbon nanotube (PAN/CNT) nanofiber films and high‐capacity water‐storage hydrogels, including both conventional and smart variants. In this integrated design, the PAN/CNT layer functions as the adsorption component, and the hydrogel layer serves as the storage component. The term BASF is used exclusively for the discussions on composite function, while the individual layers are identified by their specific compositions. Highly porous PAN/CNT nanofiber films were fabricated via electrospinning. By incorporating lithium chloride (LiCl) particles into the nanofibers, we obtained adsorption films with enhanced hygroscopic performance and ultra‐high solar absorption efficiency (Figure 2a). The PAN/CNT nanofiber layer forms a highly interconnected nanoporous network, in which nanoscale inter‐fiber voids provide abundant adsorption sites and short diffusion pathways for efficient atmospheric moisture uptake (Figure 2b). Although this nanoporous structure is optimal for vapor adsorption and release, it is not suitable for long‐term liquid water retention. In contrast, the underlying Polyacrylamide (PAM) hydrogel possesses a three‐dimensional porous matrix with interconnected open channels that enable capillary‐driven water transport and stable storage without leakage. This difference in pore structure establishes a clear structure‐function relationship, in which nanoporous nanofibers govern moisture adsorption, whereas the porous hydrogel matrix enables water storage and controlled release under solar irradiation, thereby enhancing overall water harvesting efficiency.

Fourier transform infrared spectroscopy (FT‐IR) spectroscopy confirmed the chemical composition of the BASF. Strong characteristic peaks at 3365.6 and 1633.6 cm^−1^ indicate hydroxyl (‐OH) and C‐N bonds in PAN, while the addition of CNTs slightly alters the polymer structure (Figure S1a). Raman spectra show broad peaks near 1350 and 1600 cm^−1^, evidencing the successful integration of CNTs into PAN nanofibers (Figure S1b) [35, 36]. X‐ray diffraction (XRD) analysis revealed the disappearance of PAN/CNT@15%LiCl diffraction peaks, attributed to Li^+^ coordination with polymer chains and potential hydration, enhancing hygroscopic properties [37]. Hydroxylated CNTs further improve nanofiber hydrophilicity, facilitating moisture adsorption (Figure 2c and Supplementary Movies S1 and S2). Rapid water adsorption is favored by micro‐ and mesopores with high surface area and interconnected channels, which provide abundant adsorption sites and promote fast moisture uptake. In contrast, effective water storage requires larger, stable hydrogel networks with sufficient cavity volume, preventing leakage while maintaining water retention over time. Collectively, these results underscore the critical role of hierarchical nanoporous structures in achieving rapid adsorption, robust water storage, and sustainable freshwater harvesting.

Spectroscopic and structural analyses reveal the clear structure–function complementarity of the BASF. The disappearance of characteristic diffraction peaks after LiCl incorporation indicates strong Li^+^ coordination/hydration interactions with the polymer matrix, which generate abundant hygroscopic sites for water capture. Meanwhile, the Raman peaks confirm the successful incorporation of CNTs into the PAN nanofiber framework, providing efficient solar absorption and photothermal conversion. These results demonstrate that the nanofibre layer is tailored for rapid moisture adsorption and solar‐driven regeneration, while the hydrogel layer functions as a stable water‐storage reservoir that enables leakage‐free transport, retention and controlled release of water (Figure 2d,e and Figure S1) [38]. This rationally integrated architecture not only facilitates efficient moisture capture and retention, but also imparts excellent thermal stability (Figure S2), high mechanical robustness (Figures S3–S5), and notable flexibility, which are critical for reliable operation under extreme environmental conditions (Figures S6 and S7).

The long‐term durability of the BASF was further examined by repeated adsorption‐desorption cycling. After 60 cycles, no visible macroscopic salt leakage was detected. Ionic analysis of the collected condensate further showed that Li^+^ and Cl^−^ concentrations remained low over extended operation for up to 200 cycles, with the Cl^−^concentration consistently far below the World Health Organization guideline value (Figures S8 and S9). Even under severely polluted atmospheres with PM_10_ concentrations exceeding 1500 µg·m^−3^ during continuous exposure for 5 days, the adsorption performance was only marginally affected, showing a decrease of merely 1.56%. This negligible degradation indicates that particulate accumulation does not significantly interfere with the functional adsorption sites, highlighting the excellent anti‐fouling capability of the bifunctional surface under extreme environmental conditions (Figures S10 and S11). To further evaluate long‐term durability, accelerated ultraviolet (UV) aging tests were performed, given the central role of UV irradiation in driving material degradation. Remarkably, our BASF maintains stable performance during the repeated day‐night cycling (Figure S12a) and preserve their structural and functional integrity after the simulated ten‐year UV exposure, exhibiting only a marginal performance decline of 2.6% (Figure S12b). Together, these results demonstrate that the hierarchically porous, salt‐confined and mechanically resilient architecture enables durable atmospheric water harvesting, leakage‐free water storage, solar‐assisted desorption and cost‐effective freshwater production.

### 2.2 Adsorption and Desorption Performance

**FIGURE 3:**
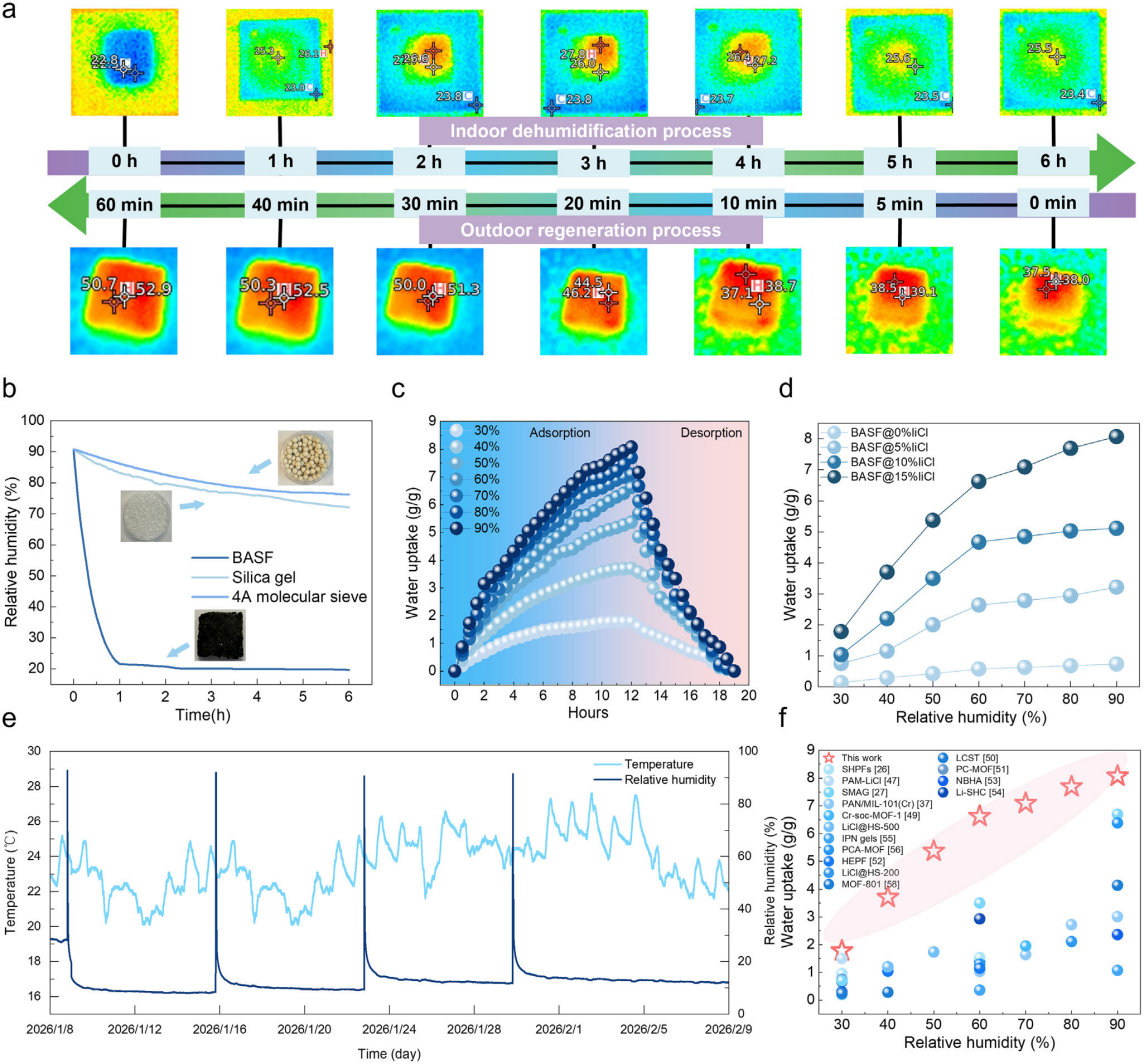
Laboratory‐scale adsorption and desorption characteristics of our BASF. (a) Infrared thermographic images capturing the thermal evolution of the BASF during dehumidification and regeneration processes. (b) Comparative evaluation of the dehumidification performance of our BASF compared with 4A molecular sieves and silica gels. (c) Moisture adsorption capacities of the BASF with varying salt loadings. (d) Adsorption and desorption behaviors of the BASF under different humidity conditions. (e) Month‐long continuous dehumidification. (f) Water harvesting performance of different adsorption materials and our BASF.

Our BASF exhibits high capability for capturing and storing water molecules, demonstrating strong potential for indoor dehumidification. During operation, water molecules in the air are first adsorbed onto active sites on the surface of the PAN/CNT films, forming a thin liquid layer. These molecules are subsequently transported through the interconnected PAN/CNT network and stored within the underlying hydrophilic hydrogel layer (Figure S13) [42, 43, 44]. Thermal imaging directly confirms the dehumidification–regeneration cycle of the BASF. During dehumidification, the film temperature rises slightly because moisture adsorption is exothermic. During regeneration, solar irradiation rapidly increases the surface temperature, accelerating water desorption and enabling near‐complete regeneration within 1 h without additional energy input. These results demonstrate the feasibility of integrating moisture adsorption with solar‐driven regeneration in a single system (Figure 3a). In addition, outstanding photothermal capability of BASF enables rapid solar‐triggered water desorption and regeneration (Figure S14).

When a small piece of our BASF (1.5 × 1.5 × 0.5 cm, approximately 1 g) was placed in a sealed container (40 × 60 × 40 cm) under high humidity (Figures S15 and S16), the relative humidity (RH) rapidly decreased from 90.7% to 21.6% within 1 h and remained low for an extended period (12 h). Such a pronounced humidity drop is fully compatible with the adsorption capacity of the BASF. Based on the chamber volume of 0.096 m^3^ and the measured RH change, the moisture in the enclosed air decreased from about 2.65 g to 0.63 g at 30°C, corresponding to a net uptake of approximately 2.02 g during the first hour. This result further highlights that the rapid RH reduction originates from the intrinsically high‐water affinity and large uptake capacity of the BASF. In contrast, 4A molecular sieves and silica gels of equivalent mass reduced the RH only from 90.7% to 86.2% and 83.3%, respectively, over the same period (Figure 3b). Thanks to the high LiCl content, our BASF achieved water adsorption capacities of 1.78 g g^−1^, 6.62 g g^−1^, and 8.07 g g^−1^ at the relative humidities of 30%, 60%, and 90%, respectively (Figure 3c), indicating strong performance across arid, semi‐arid, and humid conditions.

Efficient desorption is essential for the practical deployment of the BASF because it governs moisture release, freshwater recovery and the regeneration of active adsorption sites during cyclic operation [39, 40, 41]. A sample film was subjected to a 12‐hour water adsorption process at different relative humidities (30°C) followed by a 6‐hour desorption process (10 % RH, 60°C) in temperature and humidity controlled chamber, demonstrating rapid water release for regeneration (Figure 3d). The dehumidification performance remained highly stable over multiple adsorption‐desorption cycles (Figure 3e and Figures S17 and S18). Thermal imaging further confirmed the fast dehumidification and regeneration: surface temperatures increased during adsorption and reached approximately 50°C under outdoor conditions, enabling near‐complete water release within 1 h (Figure 3a). Additionally, the BASF was exposed to solar irradiation of varying intensities to trigger water release (Figure S19a). Under the solar heating of 1000 W m^−2^, the surface temperature rapidly rose to 67.8°C within 20 min and reached approximately 90°C within one hour (Figure S19b). Overall, our BASF exhibited superior water harvesting performance across the full range of relative humidities, compared with the previously reported materials (Figure 3f).

Conventional hydrogels typically operate through a vaporization‐condensation cycle, in which the adsorbed water is released as vapor upon heating and subsequently condensed into liquid via an external cooling process. In contrast, smart hydrogels can directly release the adsorbed water in liquid form under solar irradiation, eliminating the need for an additional condensation step. Upon heating above the lower critical solution temperature, the weakened polymer–water hydrogen bonding and enhanced hydrophobic association among nonpolar segments trigger network collapse, thereby converting the smart hydrogel from a hydrated hydrophilic state to a dehydrated hydrophobic state [50]. To prevent the shrinkage of our smart hydrogels during the practical operation and enhance water production, the optimization of preparation parameters is highly crucial. The incorporation of AM and PVA can mitigate shrinkage, but excessive addition will reduce water adsorption capacity (Figure S20 and Supplementary Tables S2). Smart hydrogel exhibits a distinct thermo‐responsive transition, strong temperature‐dependent moisture sorption, and clear adsorption‐release cycling after LiCl loading. The contact angle variation from 60.2° to 100.1° further confirms the wettability switch across the phase transition, which is beneficial for heat‐triggered water release (Figure S21). When indoor temperatures decrease, the hydrophilic smart hydrogels efficiently adsorb water molecules from the surroundings. During the daytime, they directly release liquid water to irrigate indoor plants as indoor temperatures rise. Experimental results indicate that our small‐area smart hydrogels can produce up to 40 mL of water per day while maintaining a comfortable indoor humidity.

### 2.3 Real‐World Applications in Buildings

**FIGURE 4:**
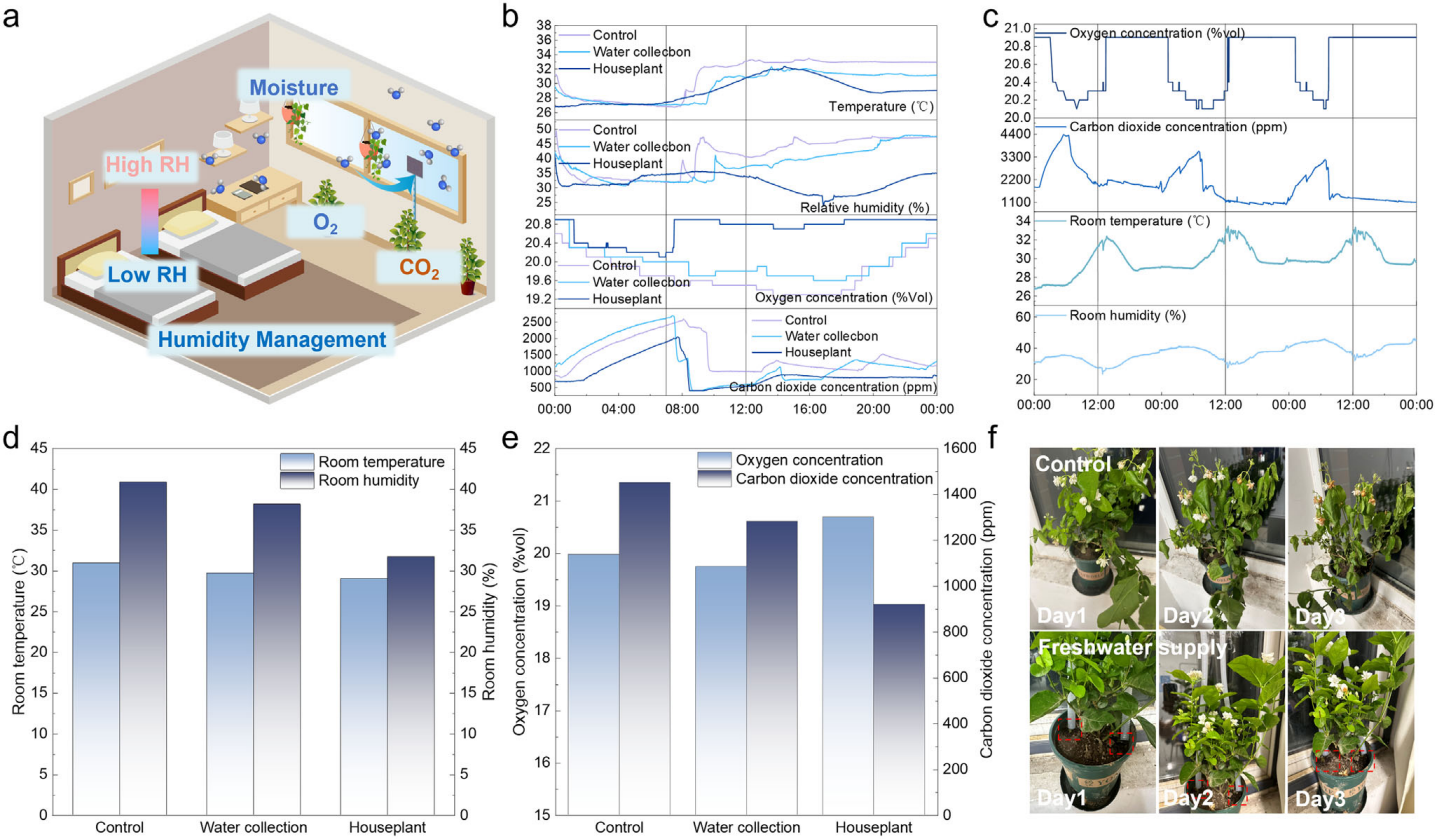
Real‐world performance of our BASF in creating a comfortable indoor living environment. (a) Schematic illustration of real‐time indoor dehumidification and freshwater collection enabled by our BASF. (b) Indoor environmental regulation performance of the BASF under different operational scenarios, including control conditions, freshwater collection, and the presence of houseplants. (c) Continuous indoor environment management performance of the BASF over three consecutive days. (d) Average indoor temperature and relative humidity under various operating conditions. (e) Oxygen and carbon dioxide concentrations measured during different operational modes. (f) Comparison of plant survival outcomes when cultivated with and without freshwater supplied by the BASF.

The BASF can harvest water from air, offering a promising approach for both indoor dehumidification and freshwater provision in buildings. To evaluate their practical performance, experiments were conducted in a bedroom setting (Figure 4a) using large‐scale BASF (25 × 28 × 1 cm) to regulate the humidity of the sealed room (3 × 5 × 3 m) (Figure S22 and Supplementary Movie S3). Within one hour, the relative humidity decreased substantially from 69.9% to 30.7% (Figure 4b). Beyond passive humidity regulation, the BASF further contributes to indoor CO_2_ management by supporting the growth of indoor plants, offering an integrated strategy for improving indoor environmental quality through coupled humidity, temperature and air‐quality control. At night, the BASF can adsorb water from indoor air, maintaining humidity within the human comfort range and thereby enhancing sleep quality (96.02%) (Supplementary Table S3) [61]. During the daytime, the harvested water is released from the hydrogel. It can either be evaporated as vapor and then condensed into liquid water for collection, or, directly exuded as liquid water for immediate plant irrigation without requiring condensation for smart hydrogels (Figures S23). Through photosynthesis, these plants can absorb CO_2_ generated by human respiration and release O_2_, further improving indoor air quality (Figure 4c).

Our BASF exerts minimal influence on indoor temperature fluctuations but effectively reduces humidity, maintaining a comfortable level (at 31.7%) over extended periods. In terms of indoor gas concentrations, the control group exhibited the highest CO_2_ levels (1451.8 ppm), far exceeding the recommended thresholds for normal working environments, while O_2_ concentrations remained relatively stable across all groups. By contrast, the rooms with indoor plants irrigated using water harvested from our BASF showed the lowest CO_2_ concentrations (920.2 ppm) and the highest O_2_ levels (20.7%, vol), demonstrating the effective regulation of indoor air quality (Figure 4d,e). Additionally, two identical potted plants were placed on windowsills, with one receiving water from the BASF and the other left untreated. The untreated plant gradually withered, whereas the plant irrigated with the harvested water exhibited normal growth, with visible soil moisture (Figure 4f). The daily water output from the BASF exceeds the typical water requirements of common indoor plants, ensuring their healthy growth (Figure 1c). Moreover, the smart hydrogel enables continuous freshwater provision without the need for additional condensation devices, supporting automatic irrigation and long‐term plant survival even during occupant absence. Overall, these results highlight the strong potential of our BASF for maintaining a healthy indoor environment and improving human well‐being.

### 2.4 Global Scalability and Impact

**FIGURE 5:**
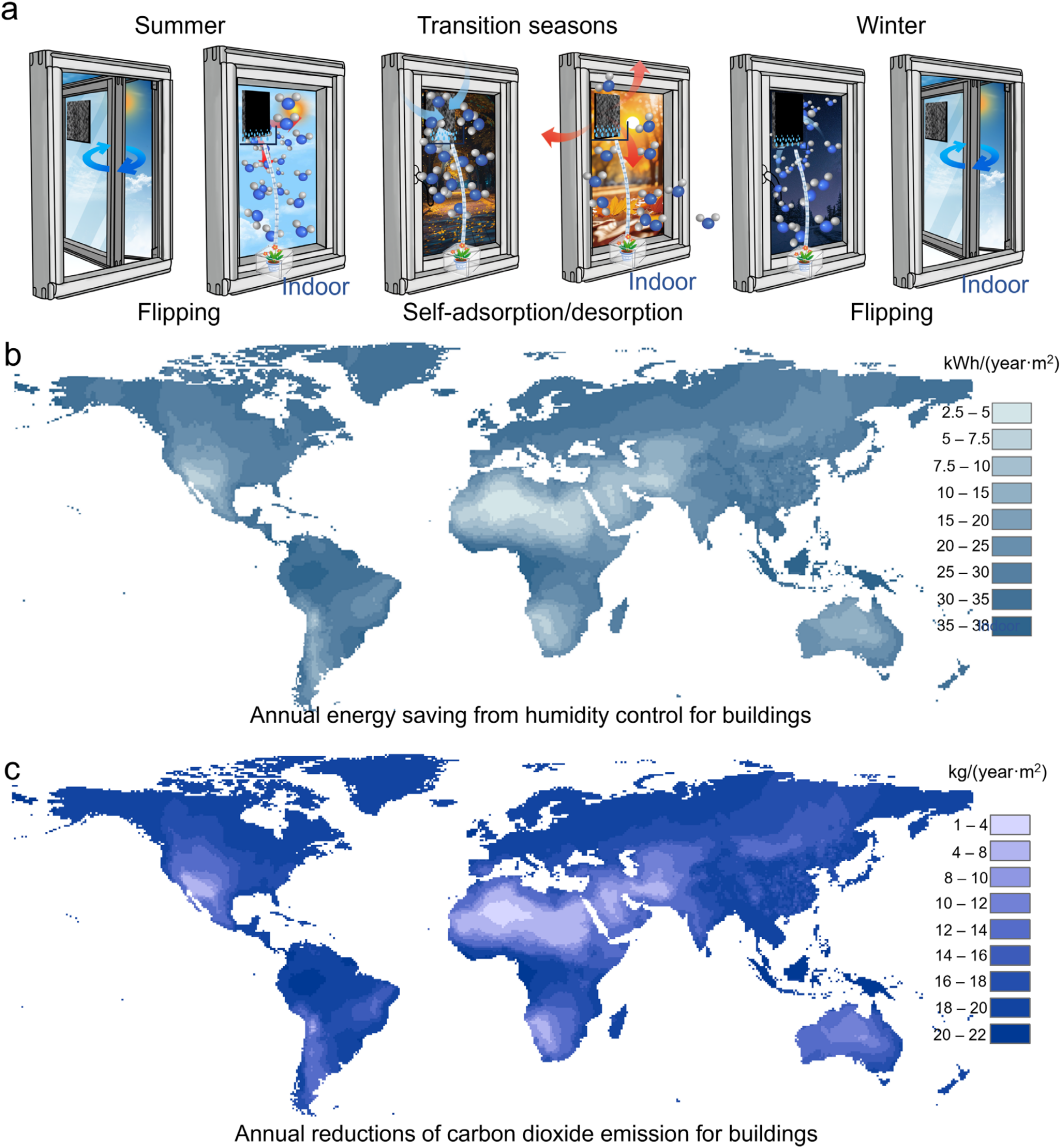
Global‐scale application potential of passive building dehumidification enabled by our BASF. (a) Schematic representation of the seasonal operation strategy for indoor humidity control and plant irrigation using the BASF. (b) Estimated global building energy savings are achievable through indoor dehumidification with the BASF. (c) Projected reductions in global CO_2_ emissions resulting from the implementation of BASF.

To evaluate its global applicability, we developed a climate‐responsive moisture‐regulation model incorporating representative meteorological datasets from different regions. In this framework, our BASF was assumed to operate primarily as a passive latent‐load regulator for buildings, with moisture adsorption occurring during humid periods and solar‐driven regeneration occurring during daytime conditions. The reduction in indoor humidity‐control demand was then translated into the dehumidification energy consumption through a conventional refrigeration‐based dehumidification model, and the corresponding CO_2_ mitigation was also estimated from the associated electricity savings. A representative 50 m^2^ building scenario was used for the global mapping analysis, and the detailed governing equations, operating assumptions, and parameter settings were provided in the Supporting Information. Indoor humidity requirements vary seasonally, and the BASF can adapt accordingly. In summer, they absorb water at night and release it outdoors during the day via solar heating. During spring and autumn, our BASF can capture moisture at night and release it indoors during dry daytime periods, maintaining the comfortable humidity ranges. In winter, they can draw moisture from outdoor air at night and release it indoors during the daytime, effectively humidifying indoor environments (Figure 5a). These seasonal operating modes can substantially reduce energy consumption associated with conventional air conditioning and lower the corresponding carbon emissions. In addition, by harvesting moisture from indoor or outdoor air at night, the BASF provides sufficient freshwater for daytime irrigation of indoor plants, thereby enabling an autonomous, maintenance‐free irrigation system.

Global simulations indicate that implementing the BASF can achieve annual energy savings of 29.9 kWh·year^−1^·m^−2^ (Figure 5b) and reduce CO_2_ emissions by 16.5 kg·year^−1^·m^−2^ (Figure 5c). The largest energy savings are predicted in tropical and subtropical regions, such as Southeast Asia, Central Africa, and South America, while substantial CO_2_ reductions are observed in densely populated warm‐humid areas, including South Asia and parts of the southern United States. These trends highlight the broad applicability and environmental benefits of the BASF for climate‐adaptive building design (Figure S24). Energy savings are particularly pronounced in high‐humidity cities; for example, Singapore and Hong Kong show reductions exceeding 20%. Beyond humidity regulation and freshwater production, the economic benefits of our BASF are notable. The material cost is only 18.79$·m^−2^ (Supplementary Table S4), and the investment can be fully recovered within 48 days in building applications. Moreover, solar‐driven regeneration eliminates operational energy consumption, substantially lowering overall building energy demand. With low initial investment and minimal operating costs, our BASF demonstrates strong market potential and are well‐positioned for commercialization and large‐scale deployment.

By integrating LiCl‐loaded nanofibers with hydrogels, the developed BASF enables rapid moisture uptake and release. Under indoor conditions, our BASF can reduce relative humidity from 90.7% to 21.6% within one hour, outperforming conventional adsorbents such as silica gels. This strategy addresses key limitations of existing adsorbents, including high energy consumption, slow response, and microbial risks. Beyond humidity control, a distinctive feature of our BASF is their ability to autonomously harvest atmospheric moisture and repurpose it for plant irrigation via an automatic watering process. Real‐world indoor experiments demonstrate that the harvested water is sufficient in both quality and quantity to support plant growth. In a controlled cultivation test, mung bean plants irrigated with the collected water exhibited root lengths (91.8 mm) and sprout growth (30.6 mm) comparable to those of plants watered with tap water, confirming the reliability of the harvested water (Figures S25 and S26). These results highlight the potential of the BASF to enable the distributed greening and urban farming, particularly in water‐stressed or high‐humidity regions.

## 3 Conclusions

In summary, our BASF provides a passive, scalable solution for indoor climate regulation and freshwater supply, addressing critical challenges in energy, water, and environmental management. By regulating humidity, harvesting water, and supporting indoor ecosystems, they offer a pathway toward sustainable urban living. Leveraging the synergistic interaction between hygroscopic LiCl particles and hydrophilic hydrogels, our BASF can rapidly reduce the relative humidity from 90.7% to 21.6% within 1 h under sealed indoor conditions. Owing to their high adsorption capacity, the average daily water yield reaches approximately 1.1 kg·m^−2^·day^−1^ under real‐world conditions. In a sealed indoor environment, the BASF adsorbs moisture at night and supply the harvested water for plant irrigation during the daytime, enabling automatic watering without manual intervention, even under unattended conditions. Photosynthetic activity further lowers indoor CO_2_ concentrations to 920.2 ppm while improving O_2_ levels for human comfort. Global modeling indicates that the BASF can achieve average annual energy savings of 29.9 kWh·year^−1^·m^−2^ and reduce CO_2_ emissions by approximately 16.5 kg·year^−1^·m^−2^. Based on the experimentally observed average water yield, the BASF can provide approximately 400 kg·m^−2^ of freshwater annually under the tested conditions, whereas the annual freshwater‐production potential could exceed 1000 kg·m^−2^ in favorable warm‐humid regions. Combined with the low material cost of 18.79$·m^−2^ and the rapid payback period of 48 days, our BASF holds strong potential for widespread adoption, offering a transformative approach to integrated humidity, water, and energy management in buildings.

The scalability of the BASF makes it promising for integration into building envelopes, such as smart windows and walls, where it could function as a passive, climate‐responsive building skin. At the system level, coupling these films with ventilation systems can reduce indoor humidity and enhance thermal comfort without additional energy input. Nevertheless, the present BASF primarily addresses humidity regulation by reducing latent loads in air‐conditioning systems. In real buildings, sensible heat, associated with air temperature control, represents a substantial portion of total energy consumption for indoor environment management. Conventional air‐conditioning systems must simultaneously manage both heat and moisture, often resulting in high energy demand and low efficiency. To further lower building energy use and carbon emissions, it is critical to develop advanced materials capable of concurrently regulating humidity and temperature across diverse indoor settings. Such coupling of passive moisture management with sensible‐heat control could provide a transformative route towards low‐carbon, climate‐adaptive buildings, especially in cities facing rising cooling demand.

## Author Contributions

J. Liu and Y. Chao contributed equally to this work. J. Liu and J. Yan conceived the idea and designed the experimental protocols. Y. Chao performed the experiments and characterization of materials with the assistance of Y. Du and C. Wang. Y. Chao, H. Wei and X. Yang performed the map modelling of this work under the guidance of Z. Zeng. Y. Chao analyzed the data and wrote the manuscript under the guidance of H. Wei, Z. Zhou, H. Yang, Z. Zeng, J. Liu, H. Cui and J. Yan. J. Liu, H. Cui and J. Yan directed the research. All authors contributed to the editing of this manuscript.

## Conflicts of Interest

The authors declare no conflict of interest.

## Supporting information




**Supporting File 1**: advs76145‐sup‐0001‐SuppMat.docx.

**Supporting File 2**: advs76145‐sup‐0002‐Movie1.mp4.

**Supporting File 3**: advs76145‐sup‐0003‐MovieS2.mp4.

**Supporting File 4**: advs76145‐sup‐0004‐MovieS3.mp4.

## Data Availability

The data that support the findings of this study are available from the corresponding author upon request.
