# Supplementary material for: Solar‐Driven Bifunctional Adsorption‐Storage Films for Ultra‐Fast Dehumidification and Freshwater Supply in Low‐carbon Buildings
Source: Adv Sci (Weinh). 2026 Jun 18:e76145. Online ahead of print. doi: 10.1002/advs.76145 (PMC13336636; doi:10.1002/advs.76145)
Supplement: Supplementary file 1 — Supporting File 1: advs76145‐sup‐0001‐SuppMat.docx. [file ADVS-9999-e76145-s001.docx]

*Supporting Information*

Solar-Driven Bifunctional Adsorption-Storage Films for Ultra-Fast Dehumidification and Freshwater Supply in Low-carbon Buildings

*Yuechao Chao, Junwei Liu*, Zhihua Zhou, Yahui Du, Hailu Wei, Haibin Yang, Xueqing Yang, Cheng Wang, Zhenzhong Zeng,* *Hongzhi Cui* and Jinyue Yan**

Y. Chao, Prof. Z. Zhou, X. Yang, C. Wang, Y. Du

School of Environmental Science and Engineering, Tianjin University, Tianjin, 300350, China.

Y. Chao, Dr. J. Liu, H. Wei, X. Yang, C. Wang, Y. Du and Prof. J. Yan

International Centre of Urban Energy Nexus, The Hong Kong Polytechnic University, Kowloon, Hong Kong, China.

Email: junweei.liu@polyu.edu.hk; j-jerry.yan@polyu.edu.hk

H. Yang and Prof. H. Cui

Key Laboratory for Resilient Infrastructures of Coastal Cities (MOE), College of Civil and Transportation Engineering, Shenzhen University, Shenzhen 518060, Guangdong, China.

Email: h.z.cui@szu.edu.cn

Dr. J. Liu, H. Wei and Prof. J. Yan

Department of Building Environment and Energy Engineering, The Hong Kong Polytechnic University, Kowloon, Hong Kong, China.

Prof. Z. Zeng

School of Environmental Science and Engineering, Southern University of Science and Technology, Shenzhen 518055, China.

**Contents**

[**Note S1 Supplemental Experimental Methods 3**](#_Toc227540346)

[**Chemical and Materials 3**](#_Toc227540347)

[**Preparation of PAN/CNT Nanofiber Membranes 3**](#_Toc227540348)

[**PAM hydrogel Preparation 3**](#_Toc227540349)

[**Preparation of Bifunctional Composite Adsorbent (PAN/CNT/Hydrogel@15% LiCl) 4**](#_Toc227540350)

[**PANPAM hydrogel Preparation 4**](#_Toc227540351)

[**Characterizations 4**](#_Toc227540352)

[**Anti-freezing Test of BASF: 5**](#_Toc227540353)

[**Salt Leakage Test of BASF: 5**](#_Toc227540354)

[**Dust Resistance Test of BASF: 5**](#_Toc227540355)

[**Prolonged UV Exposure: 5**](#_Toc227540356)

[**Interfacial Peel Test: 5**](#_Toc227540357)

[**Indoor Experiments 6**](#_Toc227540358)

[**Measurement of Moisture Absorption 6**](#_Toc227540359)

[**Photothermal conversion and solar-to-thermal energy utilization measurement of BASF: 10**](#_Toc227540360)

[**Simulation assumptions for global energy-saving and CO_2_-mitigation analysis 11**](#_Toc227540361)

[**Supplementary Tables 13**](#_Toc227540362)

[**Supplementary Figures 17**](#_Toc227540363)

[**References 42**](#_Toc227540364)

# Note S1 Supplemental Experimental Methods

### Chemical and Materials

Acrylamide monomer (AM, 99%), *N*,*N*,*N*'*N*'-tetramethylethylenediamine (TEMED, 99%), Polyacrylonitrile (PAN, *M*_W_=150000), N,N-Dimethylfomamide (DMF, 99.5%), Carbon nanotube (CNT, >95%), Polyvinyl alcohol (PVA, AR), 2-hydroxy-2-methylpropiophenone (HMPP, 99%), lithium chloride (LiCl, 99%), Ammonium persulfate (APS, 99%) were purchased from Maclin. *N*,*N*'-methylenebisacrylamide (MBA, 99%) was purchased from Aladdin. All the materials were used directly without any treatment such as purification or modification.

### Preparation of PAN/CNT Nanofiber Membranes

PAN/CNT nanofiber membranes were prepared using electrospinning technology. Specifically, 5 wt% hydroxylated carbon nanotubes were dispersed in DMF via ultrasonic treatment for 2 hours. Subsequently, 10 wt% PAN was added to the solution and mixed under magnetic stirring. The mixture was vigorously stirred for 12 hours. The PAN/CNT solution was loaded into a 5 mL syringe, with a tip-to-collector distance set at 15 cm, an applied voltage of 20 kV, and a flow rate of 1 mL·h^-1^. The environmental conditions for electrospinning were 25±1 ℃ temperature and 40±3% relative humidity, for a duration of 3 hours. Finally, the prepared black PAN/CNT with a multilayered network structure was dried at 100 ℃.

### Hydrogel Preparation

A facile one-pot method was employed to synthesize polyacrylamide (PAM) hydrogel.^[1]^ At room temperature (23 ℃), 60 g of deionized water was mixed with 12 g of AM, the mixture was stirred using a magnetic stirrer until the monomer was completely dissolved. While stirring continuously, 3 mg of MBA was added as a crosslinker, 8.52 mg of APS was added as an initiator. Finally, 7.2 µL of TEMED was added as an accelerator, the pre-gel solution was quickly transferred into a covered polypropylene square mold (dimensions: 10 cm × 10 cm), ensuring minimal air entrapment by nearly filling the mold completely. The mixture was allowed to cure overnight at room temperature, resulting in the formation of the PAM hydrogel after one day.

### Preparation of Bifunctional Composite Adsorbent (PAN/CNT/Hydrogel@15% LiCl)

To prepare the bifunctional composite adsorbent, the as-spun PAN/CNT nanofiber membrane was first fixed in the mold, followed by introduction of the PAM precursor solution. Due to the porous and interconnected structure of the PAN/CNT membrane, the precursor solution infiltrated the nanofiber network and interfacial region before gelation. After curing, the PAM hydrogel formed in situ and was anchored within the nanofiber framework, resulting in an integrated adsorption-storage architecture with intimate interfacial contact. The resulting PAN/CNT/Hydrogel composite was dried to a constant weight and then immersed in a 15 wt% LiCl aqueous solution, prepared by dissolving LiCl in deionized water under stirring for 30 min, for 24 h to achieve thorough salt impregnation.

### Characterizations

The microscopic morphologies, element analysis of PAN/CNT, PAN/CNT@15%LiCl, PAM hydrogel was observed by a field emission scanning electron microscope (SEM, FEIOUANTA FEG250). Thermogravimetric analysis (TG, NETZSCH TG 209 F3) was performed under a nitrogen atmosphere, with heating rates set at 20 °C/min across a temperature range of 24 °C to 800 °C. The water contact angles were measured by a surface tension meter (Chengde Dingsheng JY-82C video contact angle tester). The absorbance of PAN/CNT, PAN/CNT@15%LiCl were measured by a UV-visible-near infrared spectrophotometer (Shimadzu UV-3600i Plus, Japan). Raman spectra were evaluated using a Raman spectrometer (Horiba scientific, Japan). The Fourier-transform infrared spectroscopy (FT-IR) analyses were conducted with Scientific Nicolet iS20 Thermos (USA) using the ATR method, with a scanning number of 32, a wavenumber range of 400-4000 cm^-1^. The ion concentration was analyzed by inductively coupled plasma atomic emission spectroscopy (ICP, Thermo), ion chromatography (Thermo).

### Anti-freezing Test of BASF:

To assess the anti-freezing performance of BASF, samples were stored at -20 ℃ for 14 days. Mechanical integrity was subsequently assessed through tensile and torsion tests. The films were further subjected to 60 water sorption-desorption cycles, consisting of adsorption at 25 ℃ and 90% RH followed by desorption at 80 ℃ and 20% RH.

### Salt Leakage Test of BASF:

Salt leakage behavior of BASF was evaluated over 60 consecutive adsorption-desorption cycles. Each cycle consisted of adsorption at 20 ℃ and 90% RH for 12 h, followed by desorption at 80 °C and 20% RH for 6 h. After the fifth and tenth cycles, the films were placed on high-wettability, dust-free paper and photographed to visually examine potential salt residue or leakage.

### Dust Resistance Test of BASF:

The dust resistance of BASF was evaluated under simulated indoor environments with high particle concentrations. Samples were exposed to air containing PM_2.5_ and PM_10_ levels exceeding 1500 μg·m^-3^ for five consecutive days. After exposure, the amount of surface dust was measured, and scanning electron microscopy (SEM) was performed to examine the influence of dust on the film’s microstructure. The results provide insight into the antifouling properties and structural stability of the films under indoor conditions.

### Prolonged UV Exposure:

Ultraviolet aging was performed using a UVA-340 lamp following ASTM G154, with irradiance calibrated to an annual UV energy of 89,700 kJ m^-^² in Beijing, corresponding to ten years of outdoor exposure. Reflectance and transmittance were measured before and after aging to evaluate optical stability.

### Interfacial Peel Test:

The interfacial adhesion between the PAN/CNT nanofibrous membrane and the hydrogel layer was characterized using a YC-BL-A peel tester (Yicheng, China). Rectangular specimens (10 mm in width and ~80 mm in length) were prepared, and a free membrane tab (~15 mm) was reserved for gripping. The hydrogel side was fixed in the lower clamp, while the free end of the nanofibrous membrane was attached to the upper clamp. Peeling was performed at a constant rate of 30 mm min^-1^ under an approximately 180° configuration. Force-time curves were continuously recorded during the test.

### Indoor Experiments

The indoor experiment was conducted in Tianjin University (China 38.99° N, 117.31° E). The bifunctional composite adsorbent was affixed to the window, a customized plant cultivation apparatus was placed around it. The room was then sealed as tightly as possible to minimize airflow. The two experimenters consistently woke up at 7:30 AM. One left the dormitory while the other remained until 12:00 PM, at which point they also departed. Both experimenters returned to the dormitory at 11:30 PM to rest. This routine was maintained for two consecutive days. On the third day, neither experimenter returned to the dormitory after waking. Outside of these specified periods, the experimenters did not occupy the room. Hourly weight changes of the adsorbent were recorded using a gravity sensor. Oxygen and carbon dioxide concentrations within the room were continuously monitored using respective sensors. The indoor temperature was monitored using a thermocouple under light-shielding conditions, continuous data collection was performed using the RX6032C data acquisition device. The humidity inside the room was recorded using a humidity logger.

### Measurement of Moisture Absorption

The moisture adsorption capacity, cyclic stability was assessed in a controlled temperature, humidity chamber. The moisture adsorption test was performed at 30 °C under various relative humidity levels (30 %, 40 %, 50 %, 60 %, 70 %, 80 %, 90 %). Prior to testing, the samples were cut into uniform squares measuring 1.5×1.5×0.5 cm, dried in an oven at 100 °C until a constant weight was achieved, subsequently placed in the chamber. Samples were periodically removed (every 30 minutes), promptly weighed using an electronic balance with a precision of 0.0001 g. For the moisture adsorption-desorption cyclic test, samples were maintained at 30 °C, 60% RH for 12 hours to adsorb moisture, followed by 6 hours at 60 °C to facilitate water vapor desorption. The moisture adsorption capacity of the samples was calculated according to the following equation:

$C_{abs}=\frac{\triangle m}{m_{0}}$ (1)

In this equation, *C*_abs_ denotes the moisture adsorption capacity per unit weight (g·g^-1^), Δ*m* represents the mass of adsorbed moisture (g), *m*_0_ represents the initial mass of the dry material (g).

The swelling rate experiment of smart hydrogels was conducted in a sealed container. Hydrogels of the same size, weight were placed in a transparent sealed container (10×10×10 cm), with 100 ml of deionized water added to ensure the complete immersion of smart hydrogels. After soaking for 1, 2, 4, 8, 12, 16, 20, 24, 28 hours, the samples were removed, the residual surface water was immediately wiped off, the samples were weighed using an electronic balance with an accuracy of 0.0001 g. After measurement, the samples were returned to the sealed container to continue the swelling experiment.

The water adsorption capacity of the samples was calculated using the following formula:

$C_{\text{water}}=\frac{\triangle m}{m_{0}}$ (2)

In this equation, *C*_water_ represents the water adsorption capacity per unit weight (g·g^-1^).

First, the relationship between indoor, outdoor temperature, humidity was established. The calculation formula is as follows:^[2],[3]^

$AH=\frac{1322.7}{T+273.15}\times e^{(\frac{17.625\times T}{T+243.04})}\times\frac{RH}{100}$ (5)

In the equation, AH represents the absolute humidity (%).

The mathematical model for the transfer of moisture, heat in air is established using the following equations.

The mass conservation equation for water molecules：

$\frac{\partial Y}{\partial t}\text{d}t\times(A\rho_{\text{A}}\text{d}x)+\frac{\partial Y}{\partial x}\text{d}x\times(VA\rho_{\text{A}}\text{dt})+\frac{\partial W}{\partial t}\text{d}t\times(f_{d}\text{d}x)=0$ (6)

In the equation, the first term represents the change in the mass of water adsorbed/desorbed by the processed/regenerated air over a time interval *d*_t_. The second term represents the change in the moisture content of the air between the inlets and outlet. The third term represents the change in the mass of water adsorbed/desorbed by the adsorbent over the time interval *d*_t_. *Y* represents the moisture content in the air (g·kg^-1^ dry air), *A* is the cross-sectional area (m²), *ρ*_A_ is the air density (kg·m^-3^), *V* is the air velocity in the adsorption/desorption channel (m·s^-1^), *W* is the amount of water adsorbed by the adsorbent (g·kg^-1^), *f*_d_ is the mass of the adsorbent material along the airflow direction (kg·m^-1^).

The equation for the velocity of water molecule transfer:

$\frac{\partial W}{\partial t}\text{d}t\times(f_{d}\text{d}x)=K_{\text{Y}}\text{(Y-Y}_{\text{W}}\text{)}P\text{d}t\text{d}x$ (7)

In the equation, *K*_Y_ is the mass transfer coefficient of water vapor at the adsorbent surface (kg·m^-2^·s^-1^), *P* is the perimeter of the section (m), *W*_max_ is the maximum adsorption capacity of water vapor on the adsorbent (g·kg^-1^), *Y*_W_ is the moisture content of the air when adsorption equilibrium is reached between the adsorbent, the air (g·kg^-1^).

The energy conservation equation:

$\begin{matrix} (YC_{\text{pv}}+C_{\text{pa}})\frac{\partial T_{k-air}}{\partial t}\text{d}t\times A\rho_{A}\text{d}x+(YC_{\text{pv}}+C_{\text{pa}})\frac{\partial T_{k-air}}{\partial x}\text{d}x\times VA\rho_{A}\text{d}t \\ +(C_{\text{pd}}+WC_{\text{pl}})\frac{\partial T_{k-adsorbent}}{\partial t}\text{d}t\times f_{\text{d}}\text{d}x+C_{\text{pm}}\frac{\partial T_{k-adsorbent}}{\partial t}\text{d}t\times f_{\text{m}}\text{d}x \\ =q\frac{\partial W}{\partial t}\text{d}t\times f_{\text{d}}\text{d}x \end{matrix}$ (8)

In the equation, the first term represents the energy change of air during the adsorption/desorption process over the time interval *d*_t_. The second term represents the energy change of air between the inlet and outlet during *d*_t_. The third term represents the energy change of the adsorbent during the adsorption/desorption process over *d*_t_. The fourth term represents the energy change of the substrate during the adsorption/desorption process over *d*_t_. The fifth term represents the heat of adsorption released during the adsorption of water by the adsorbent. *T*_k-aie_ is the absolute temperature of the air (K), *T*_k-adsorbent_ is the absolute temperature of the adsorbent (K), *f*_m_ is the mass of the substrate along the airflow direction (kg·m^-1^), *C*_pa_ is the specific heat capacity at constant pressure of the processed air (J·kg^-1^·K^-1^), *C*_pd_ is the specific heat capacity at constant pressure of the regenerated air (J·kg^-1^·K^-1^), *C*_pl_ is the specific heat capacity at constant pressure of the water vapor adsorbed by the adsorbent (J·kg^-1^·K^-1^), *C*_pm_ is the specific heat capacity at constant pressure of the substrate (J·kg^-1^·K^-1^), *C*_pv_ is the specific heat capacity at constant pressure of the water vapor in the air (J·kg^-1^·K^-1^), *q* is the heat of adsorption of the water vapor (J·kg^-1^·K^-1^).

The heat transfer equation between air, the adsorbent:

$\begin{matrix} \frac{\partial T_{k-adsorbent}}{\partial t}\text{d}t\left[(C_{\text{pd}}+WC_{\text{pl}})f_{\text{d}}\text{d}x+C_{\text{pm}}f_{\text{m}}\text{d}x\right] \\ +\alpha P(T_{k-adsorbent}-T_{k-air})\text{d}t\text{d}x \\ +qK_{\text{Y}}P(Y_{\text{W}}-Y)\text{d}t\text{d}x=0 \end{matrix}$ (9)

In the equation, the second term represents heat transferred between the air, the adsorbent after the time interval *d*_t_ during adsorption/desorption. The third term represents the heat of adsorption released by water vapor adhering to the adsorbent after the time interval *d*_t_ during adsorption. α is the heat transfer coefficient (W·m^-2^·K^-1^).

The calculation formula for the heat of moisture adsorption:

$q=h_{\text{V}}\text{(1.0+0.2843e}^{\text{-10.28W}}\text{)}$ (10)

In the equation, *h*_V_ is the latent heat of vaporization of water vapor (J·kg^-1^).

The calculation formula for the saturation vapor pressure of water vapor in the air when the adsorbent reaches equilibrium:^[4]^

$\ln P_{\text{WS}}\text{=23.196-}\frac{3816.44}{T_{\text{W}}\text{-46.13}}$ (11)

In the equation, P_WS_ represents the saturation vapor pressure of water vapor in the air when the adsorbent reaches equilibrium (Pa).

The moisture dissipation from the human body should also be considered indoors. The moisture dissipation rate for an adult male can be directly referenced in Supplementary Table 1.

The calculation of moisture exchange is as follows:

$\text{d}W=G\text{d}d=\beta(P_{q}-p_{qb})\text{d}F$ (12)

In this equation, *G* represents the amount of air in contact with water (kg·s^-1^), *β*is the moisture exchange coefficient between air, water surface calculated based on the water vapor partial pressure difference (kg·N^-1^), *P*_q,_ *P*_qb_ denote the water vapor partial pressures of the bulk air, the boundary layer air (Pa).

The cooling capacity of the refrigeration dehumidifier is:

$Q_{0}=G(h_{1}-h_{2})$ (13)

The dehumidification capacity is:

$W=G(d_{1}-d_{2})$ (14)

The heat dissipation capacity of the condenser is:

$Q_{k}=G(h_{3}-h_{2})$ (15)

The thermal equilibrium of the refrigeration system is:

$\begin{matrix} Q_{k}=Q_{0}+N_{i} \\ G(h_{3}-h_{2})=G(h_{1}-h_{2})+N_{i} \end{matrix}$ (16)

In this equation, *N*_i_ represents the input power of the refrigeration compressor (kW).

The moisture content of the air after the evaporator is:

$d_{2}=d_{1}-\frac{W}{G}$ (17)

### Photothermal conversion and solar-to-thermal energy utilization measurement of BASF:

The photothermal performance of BASF, a bilayer membrane composed of an upper PAN/CNT nanofibrous photothermal layer and a lower PAM hydrogel layer, was evaluated under simulated solar irradiation. The sample was cut into a square shape with a lateral size of 5×5 cm^2^ and irradiated at an intensity of 1 sun (1000 W m^-2^). During illumination, the temperatures of the top surface, interfacial region, and bottom surface were recorded simultaneously using thermocouples to monitor the heat generation and through-thickness heat transfer behavior of the bilayer membrane.

The apparent solar-to-thermal storage efficiency of BASF was estimated from the sensible heat stored in the membrane according to:

$\eta_{store}=\frac{\sum m_{i}c_{i}\Delta T_{i}}{IAt}$ (18)

where *m*_i_, *c*_i_, and *ΔT*_i_ are the mass, specific heat capacity, and temperature rise of each layer, respectively, *I* is the incident solar intensity, *A* is the illuminated area, and *t* is the irradiation time. For the bilayer BASF membrane, the stored heat was mainly contributed by the PAM hydrogel layer, while the heat capacity contribution of the ultrathin PAN/CNT nanofibrous layer was negligible. Therefore, the average temperature rise of the hydrogel layer was approximated from the interfacial and bottom temperatures.

Considering that water evaporation occurred simultaneously during solar irradiation, the overall apparent solar-to-thermal energy utilization efficiency was further estimated by including both sensible heat storage and evaporation enthalpy:

$\eta_{\text{total}}=\frac{Q_{stored}+Q_{evap}}{IAt}$ (19)

where *Q*_stored_ is the stored sensible heat in the bilayer membrane and *Q*_evap_ is the heat consumed by water evaporation, calculated as:

$Q_{evap}=m_{evap}L_{v}$ (20)

with *m*_evap_ being the mass loss of water during irradiation and *L*_v_ the latent heat of water evaporation.

In addition, the through-thickness temperature gradient of BASF was analyzed from the recorded top, interface, and bottom temperatures to reveal the heat transfer characteristics between the photothermal PAN/CNT layer and the hydrogel substrate. This method allows evaluation of not only the photothermal heating capability of BASF, but also its coupled thermal storage and evaporation behavior under solar illumination.

### Simulation assumptions for global energy-saving and CO_2_-mitigation analysis

For the global simulation, a representative air-conditioned residential building with a floor area of 50 m^2^ was selected. In this study, BASF was assumed to serve as a passive indoor humidity-management material, mainly reducing the latent load associated with indoor moisture control, while the sensible heat load was still handled by the conventional air-conditioning system. Accordingly, the reference case was defined as a building using a refrigeration-based air-conditioning/dehumidification system, and the reduction in indoor dehumidification demand achieved by BASF was converted into avoided electricity consumption based on the refrigeration model described in Equations (13)-(17).

The simulation was carried out using typical meteorological data from different global regions. Seasonal operation of BASF followed the working modes illustrated in Figure 5a. In summer, BASF was assumed to absorb moisture during humid nighttime periods and regenerate under daytime solar irradiation. In spring and autumn, BASF was assumed to absorb moisture at night and release moisture indoors during the daytime to maintain the indoor humidity within a comfortable range. In winter, BASF was assumed to absorb moisture from outdoor air and release it indoors during the daytime, thereby providing passive humidification. Under all cases, BASF was considered to regulate indoor humidity without additional energy input for regeneration, as the regeneration process was driven by available daytime solar irradiation.

In the global calculation, the installation of BASF was normalized to the building floor area, so that the annual energy-saving potential and CO_2_-emission reduction could be expressed on a unit-floor-area basis. The indoor moisture-load reduction induced by BASF was evaluated using the humidity-transfer, mass-conservation, and energy-conservation relationships described in Equations (5)-(12). The corresponding reduction in electricity demand for conventional dehumidification was then determined using Equations (13)-(17), and the associated CO_2_-emission reduction was further estimated from the avoided electricity consumption. Therefore, the reported values represent the potential contribution of BASF to building-scale humidity management under representative climatic conditions.

Long-term performance degradation of BASF was not explicitly introduced into the annual simulation. This assumption was adopted because the material showed stable cyclic performance during repeated adsorption-desorption tests and only a slight decline after prolonging UV aging in this work. Therefore, the simulation mainly reflects the intrinsic application potential of BASF for passive humidity regulation and building energy saving, rather than a degradation-coupled lifetime prediction.

**Supplementary Tables**

**Supplementary Table 1.** Comparison of different hygroscopic materials.

| Name | Major processed  materials | Hygroscopic property | Dehumidification performance | Single atmospheric water harvesting performance | References |
| --- | --- | --- | --- | --- | --- |
| PCLG | Hydrogel | 1.54 g/g (56% RH) | - | 2.9 L/m^2^ | [5] |
| COF-SO_3_H | COF | 0.12 g/g (10% RH)  0.3 g/g (90% RH) | - | - | [6] |
| MFBHs | Hydrogel | 0.86 g/g (15% RH)  1.32 g/g (30% RH)  2.18 g/g (60% RH) | - | 14.19 kg/kg | [7] |
| BMA | Hydrogel | 3.96 g/g (90% RH) | - | - | [8] |
| MIL-160 | MOF | 0.34 g/g (30%) | - | - | [9] |
| CaCl_2_-PAAm hydrogel | Hydrogel | - | - | 98.09 g/m^2^ | [10] |
| PAMPS | Hydrogel | 0.62 g/g (60% RH) | - | - | [11] |
| N-MAG | Hydrogel | 1.59 g/g (50% RH) | 66.9% (1h) | - | [12] |
| MOF-801 | MOF | 0.18 g/g (10% RH)  0.22 g/g (15% RH) | - | - | [13] |
| DMOFs | MOF | 0.45 g/g (90% RH) | 28.2% (3h) | - | [14] |
| Photothermal wood | Wood | 2.30 g/g (80% RH) | 28% (1h) | 4.34 kg/m^2^ | [15] |
| MAWH | Aerogel | 3.12 g/g (90% RH)  2.65 g/g (70% RH)  1.90 g/g (50% RH)  1.14 g/g (30% RH) | 54% (6h) | 1.02 g/g | [16] |
| SHC | Polymer | 4.64 g/g (95% RH)  3.04 g/g (90% RH)  1.87 g/g (80% RH)  1.15 g/g (70% RH)  0.61 g/g (60% RH) | 18% (15min) | - | [17] |
| ABMTF | Fabric | 2.05 g/g (90% RH)  1.73 g/g (80% RH)  1.31 g/g (70% RH)  0.96 g/g (60% RH) | 15% (1h) | - | [18] |
| CMCS-Ca@SF | Aerogel | 1.35 g/g (90% RH)  0.66 g/g (70% RH)  0.27 g/g (50% RH) | 25% (5h) | - | [19] |
| o-COF | COF | 0.54 g/g (93% RH) | 30% (6h) | - | [20] |
| PAN/BPA_2_ | Polymer | 2.65 g/g (90% RH)  1.02 g/g (80% RH)  0.18 g/g (70% RH) | 20.7% (1h) | - | [21] |
| Fe-Co hydrogel | Hydrogel | 5.22 g/g (95% RH)  3.62 g/g (90% RH)  2.91 g/g (85% RH)  2.01 g/g (80% RH)  1.76 g/g (75% RH)  1.48 g/g (70% RH)  1.29 g/g (60% RH)  1.02 g/g (50% RH)  0.90 g/g (40% RH) | 23.8% (0.3h) | 1.8 g/g | [22] |
| NBHA | Aerogel | 2.36 g/g (95% RH)  0.30 g/g (35% RH) | - | 0.37 g/g | [23] |
| PAM-MXene | Hydrogel | 5.86 g/g (90% RH)  4.29 g/g (80% RH)  2.36 g/g (60% RH)  1.50 g/g (40% RH)  1.00 g/g (30% RH) | - | 1.125 g/g | [24] |
| LiCl@MIL-101(Cr)) | MOF | 0.77 g/g (30% RH) | - | 0.45 g/g | [25] |
| SCAC | Aerogel | 0.12 g/g (80% RH)  0.07 g/g (60% RH)  0.05 g/g (40% RH) | - | 0.57 g/g | [26] |
| ILCA | Polymer | 1.20 g/g (60% RH)  0.09 g/g (30% RH) | - | 0.563 g/g | [27] |
| D-Sorbents | Hydrogel | 0.81 g/g (80% RH) | - | 0.54 g/g | [28] |
| MIL-101(Cr)/Fc(COOH) | MOF | 1.04 g/g (80% RH)  0.93 g/g (60% RH) | - | 0.71 g/g | [29] |
| PAMg-Li | Complex | 1.216 g/g (50% RH)  0.974 g/g (30% RH) | - | 1.403 g/g | [30] |
| PCC-42 | MOF | 0.90 g/g (50% RH)  0.76 g/g (40% RH)  0.59 g/g (30% RH)  0.45 g/g (20% RH) | - | 0.4 g/g | [31] |
| PDA@PP-Cl | Hydrogel | 2.76 g/g (90% RH)  2.47 g/g (80% RH)  1.92 g/g (70% RH)  1.61 g/g (60% RH)  0.68 g/g (30% RH) | - | 1.31 g/g | [32] |
| CNF/LiCl-1.5 | Aerogel | 1.59 g/g (95% RH)  1.48 g/g (75% RH)  1.15 g/g (57% RH)  0.73 g/g (33% RH)  0.33 g/g (11% RH) | - | 0.63 g/g | [33] |
| SPHN | Nanocapsule | 2.01 g/g (80% RH)  1.40 g/g (60% RH)  0.78 g/g (30% RH) | - | 1.2 g/g | [34] |
| SMAG | MOF | 6.70 g/g (90% RH)  3.50 g/g (60% RH)  0.70 g/g (30% RH) | - | - | [35] |
| Li-SHC | Complex | 2.93 g/g (60% RH)  1.79 g/g (30% RH)  1.18 g/g (15% RH) | - | 1.09 g/g | [36] |
| LCST | Hydrogel | 0.6 g/g (80% RH) | - | - | [37] |
| PC-MOF | Polymer | 3.01 g/g (90% RH)  1.30 g/g (60% RH)  0.75 g/g (30% RH) | - | - | [38] |
| PAM-LiCl | Hydrogel | 1.5 g/g (30% RH)  1.1 g/g (20% RH) | - | 0.51 g/g | [39] |
| PAN/MIL NFM | MOF | 3.01 g/g (90% RH)  2.72 g/g (80% RH)  1.64 g/g (70% RH)  1.03 g/g (60% RH) | 11.7% (2h) | - | [40] |
| Bifunctional adsorbent films | Hydrogel | 8.07 g/g (90% RH)  7.69 g/g (80% RH)  7.09 g/g (70% RH)  6.62 g/g (60% RH)  5.36 g/g (50% RH)  3.70 g/g (40% RH)  1.78 g/g (30% RH) | 70.092 % (1h) | 4.73 g/g | Our work |

Compared with representative recent systems summarized in Supplementary Table 1, our BASF exhibits higher adsorption capacities over a wide humidity range, reaching 8.07 g g^-1^ at 90% RH, 6.62 g g^-1^ at 60% RH, and 1.78 g g^-1^ at 30% RH, while also achieving 70.092% dehumidification within 1 h. Unlike most previous studies centered on material-level atmospheric water harvesting, this work further demonstrates real-room humidity regulation, window-mounted deployment, autonomous freshwater reuse for plant irrigation, and system-level analysis of building energy and carbon benefits.

**Supplementary Table 2.** Smart hydrogels with different preparation parameters.^[41]^

| Compositions | Sample codes | | | | | | | | |
| --- | --- | --- | --- | --- | --- | --- | --- | --- | --- |
|  | Smart-1 | Smart-2 | Smart-3 | Smart-4 | Smart-5 | Smart-6 | Smart -7 | Smart -8 | Smart -9 |
| Base-material | 2.372 | 2.372 | 2.372 | 2.372 | 2.372 | 2.372 | 2.372 | 2.372 | 2.372 |
| MBA | 0.044 | 0.044 | 0.044 | 0.044 | 0.044 | 0.044 | 0.044 | 0.044 | 0.044 |
| PVA | 0.264 | 0.528 | 0.792 | 1.056 | 1.320 | 1.056 | 1.056 | 1.056 | 1.056 |
| AM | 0.240 | 0.240 | 0.240 | 0.240 | 0.240 | 0.480 | 0.720 | 0.960 | 1.20 |

**Supplementary Table 3.** Indoor environment of human sleep under different conditions.^[42]^

| Influence parameters | Sleep quality (%) | | |
| --- | --- | --- | --- |
|  | Control | Water collection | Houseplant |
| Carbon dioxide concentration (ppm) | 94.9366 % | 95.9574 % | 96.0183 % |
| Room relative humidity (%) | 98.2190 % | 99.4720 % | 99.4960 % |
| Room temperature (℃) | 99.2752 % | 99.5856 % | 99.8096 % |

**Supplementary Table 4.** Production costs of our BASF.

| Materials | Usage amount | Price | Subtotal price |
| --- | --- | --- | --- |
| LiCl | 0.08 kg/ m^2^ | 13 $/kg | 1.04 $/m^2^ |
| PAM | 0.08 kg/ m^2^ | 0.035 $/g | 2.8 $/m^2^ |
| TEMED | 72 mL/ m^2^ | 0.088 $/mL | 6.34 $/m^2^ |
| APS | 8.52 g/ m^2^ | 0.011 $/g | 0.09 $/m^2^ |
| N,N'-Methylenebis(2-propenamide) | 3 g/ m^2^ | 0.06 $/g | 0.18 $/m^2^ |
| PAN | 5 g/ m^2^ | 0.306 $/g | 1.53 $/m^2^ |
| DMF | 45 ml/ m^2^ | 0.057 $/ml | 2.57 $/m^2^ |
| CNT | 5 g/ m^2^ | 0.848 $/g | 4.24 $/m^2^ |
| Total price | 18.79 $/m^2^ | | |

**Supplementary Figures**


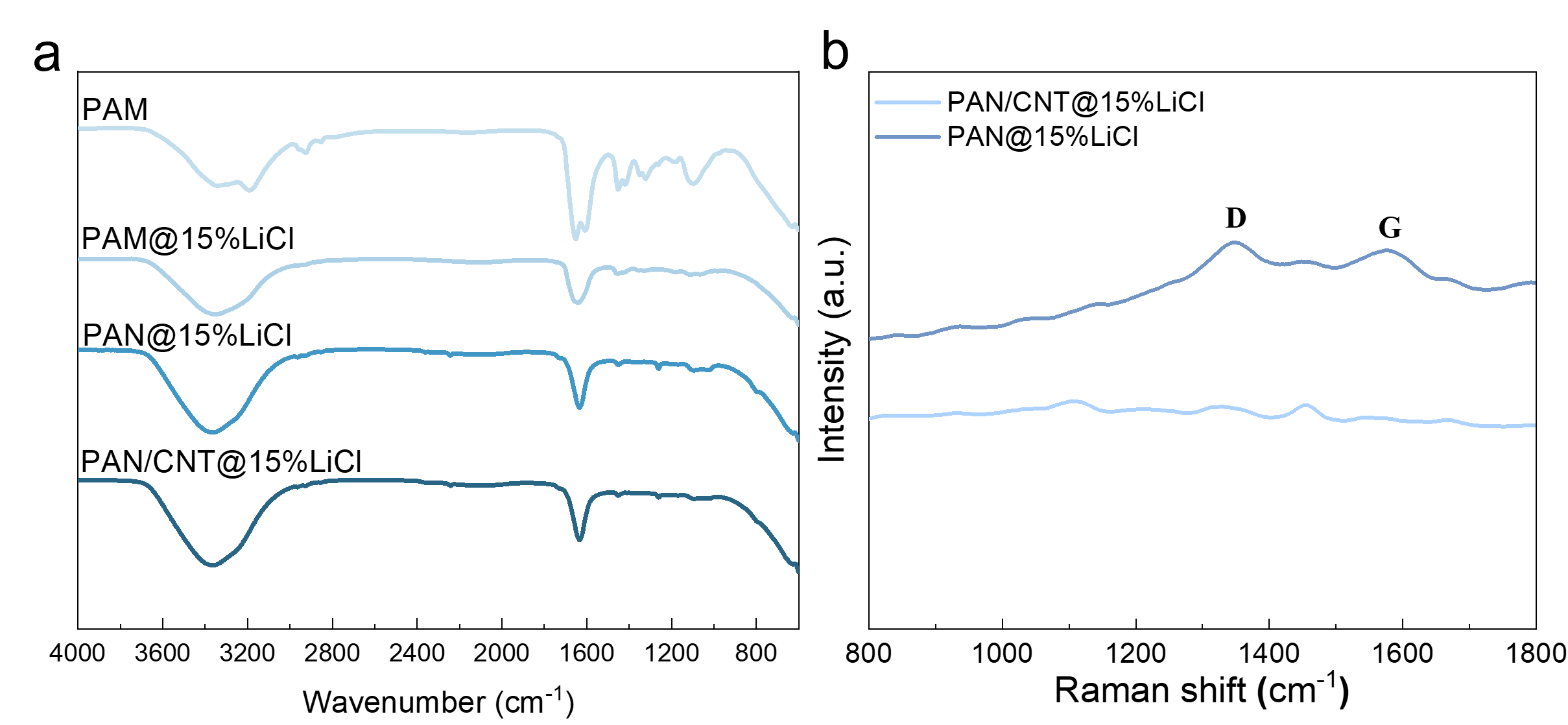


**Figure S1.** **(a)** The Fourier transform infrared spectroscopy (FT-IR) of PAM hydrogel, PAM@15%LiCl, PAN@15%LiCl and PAN/CNT@15%LiCl. **(b)** Raman spectra of PAN/CNT and PAN nanofiber membrane.^[43]^

**Figure S2.** Thermal stability of PAN@15%LiCl, PAN/CNT@15%LiCl and PAM@15%LiCl.


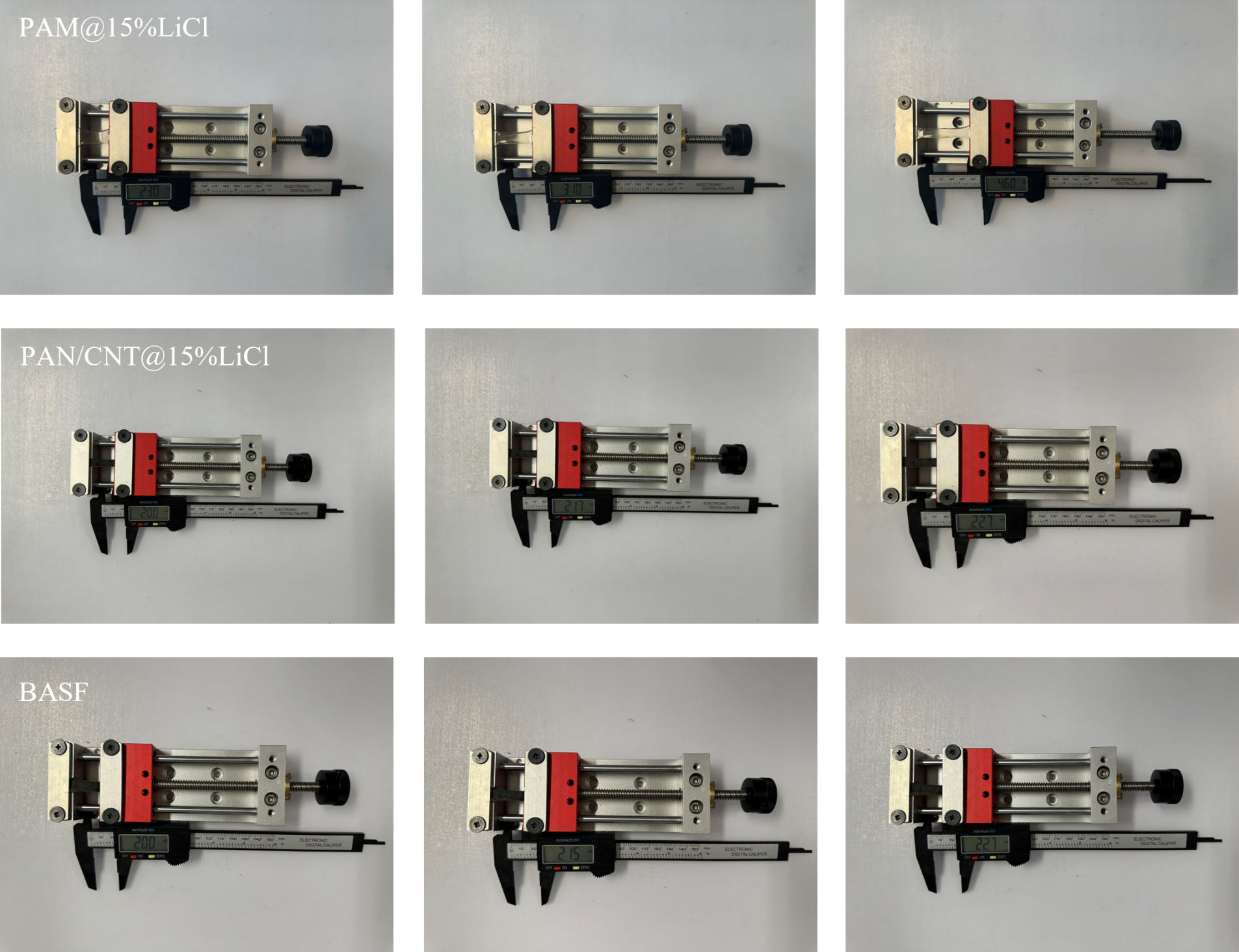


**Figure S3.** Tensile properties of PAM@15%LiCl, PAN/CNT@15%LiCl and BASF.


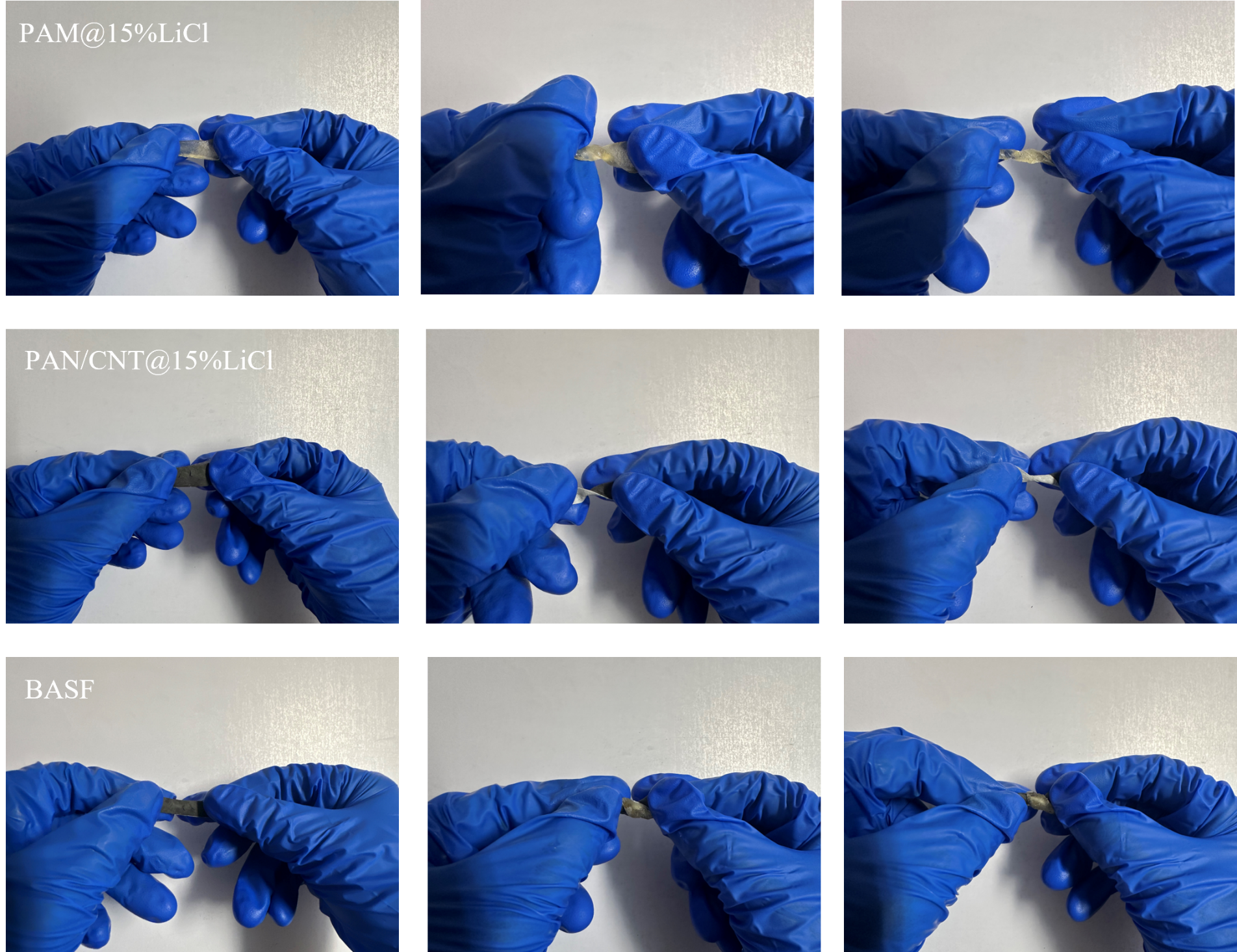


**Figure S4.** Torsional properties of PAM@15%LiCl, PAN/CNT@15%LiCl and BASF.


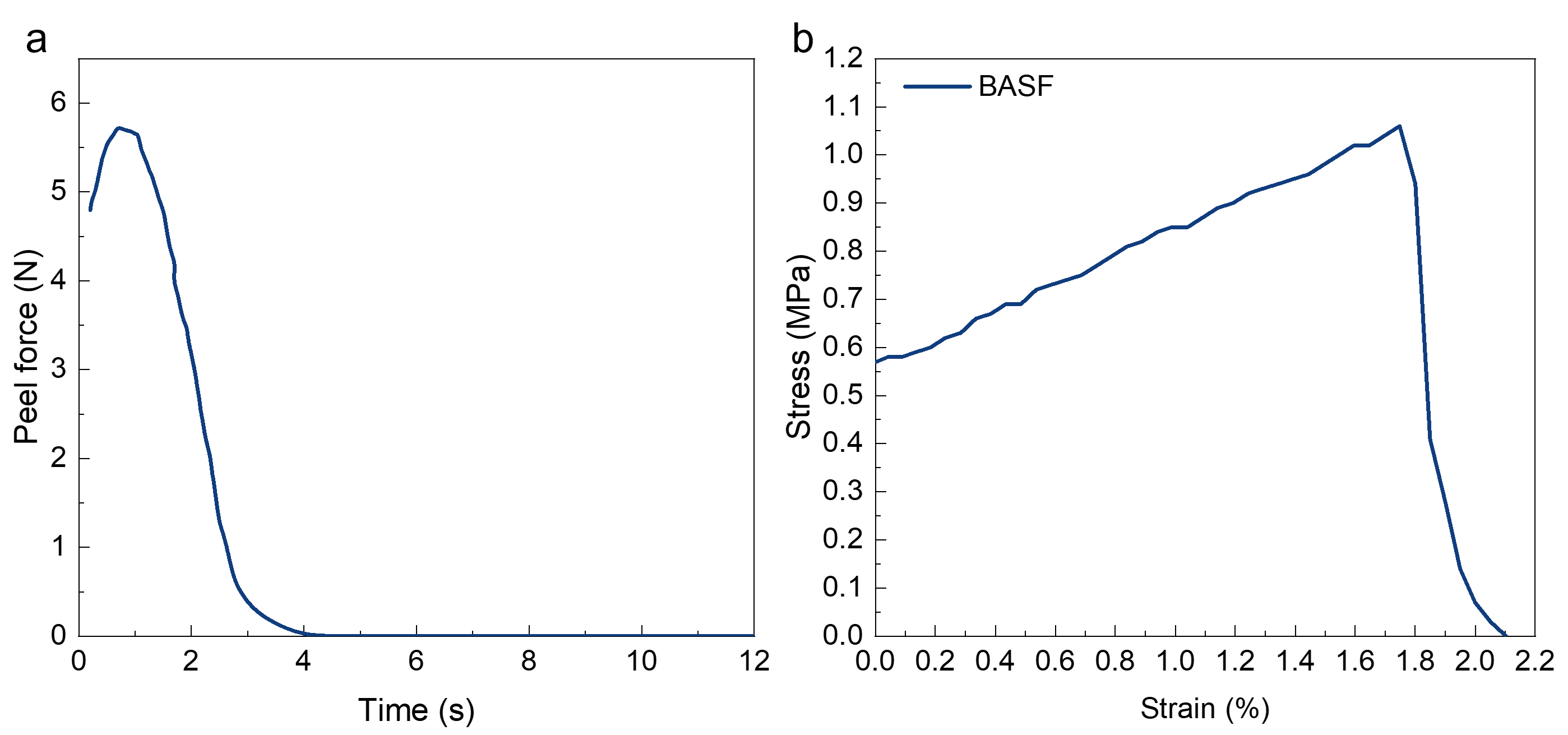


**Figure S5.** Peel and tensile mechanical properties of BASF. **(a)** Schematic illustration of the 180° peel test of BASF. **(b)** Tensile stress-strain curve of BASF.

Figure S5 illustrates the peel and tensile mechanical properties of the BASF. The 180° peel test (Figure S5a) was used to evaluate the interfacial adhesion between the PAN/CNT nanofiber layer and the PAM hydrogel layer, confirming that the two components form an integrated structure rather than a loosely stacked bilayer. The tensile stress-strain curve (Figure S5b) shows that the BASF can sustain progressively increasing stress before fracture, indicating satisfactory mechanical strength and structural integrity. The sharp drop after the maximum stress corresponds to final failure of the composite, suggesting that the BASF maintains integrated load-bearing behavior before rupture. These results collectively verify the robust interfacial bonding and mechanical stability of the BASF.


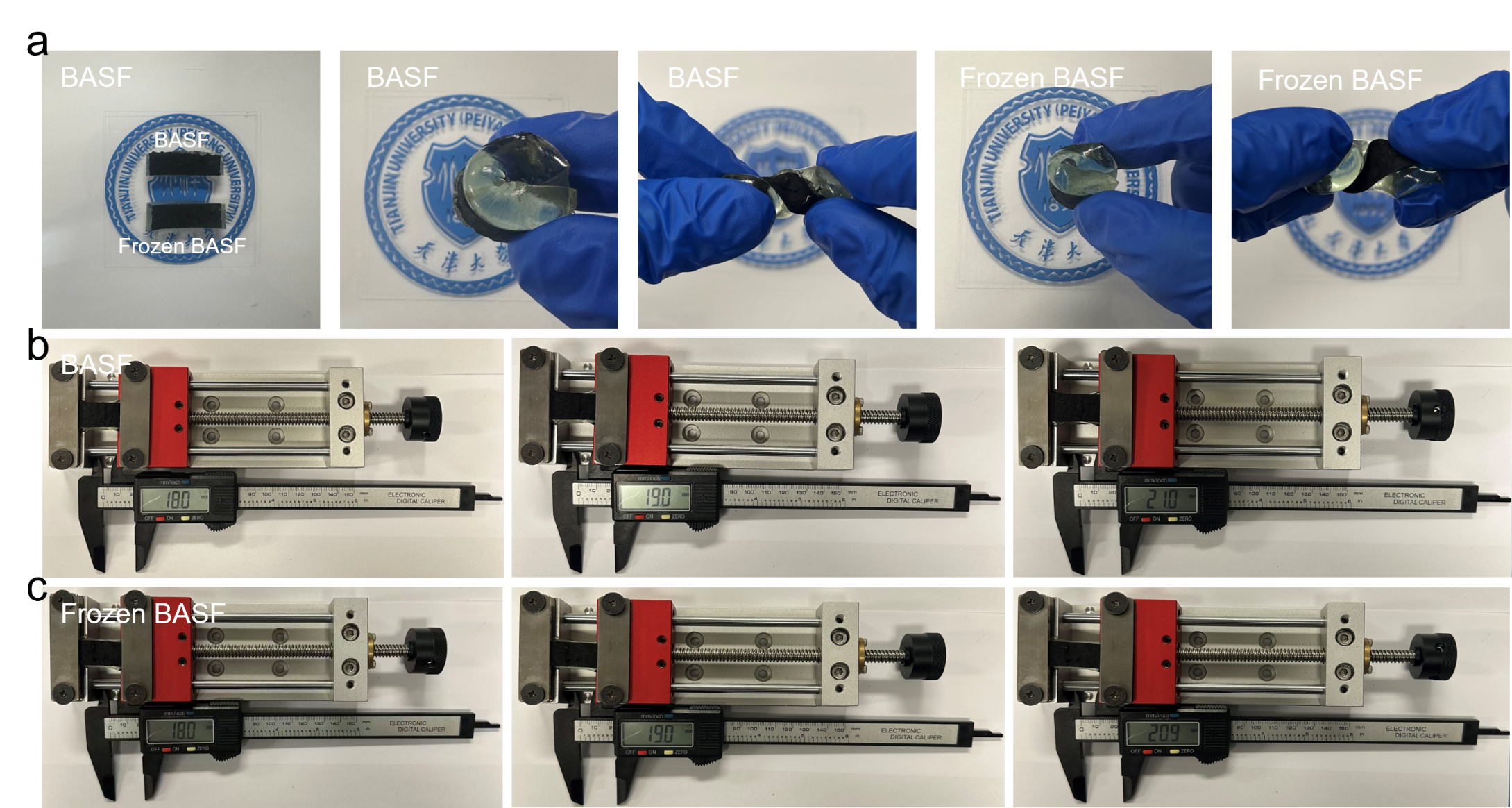


**Figure S6.** **(a)** Bending performance of the BASF after 336 h of exposure at -20 ℃. **(b)** Tensile properties of BASF. **(c)** Tensile performance of the BASF after 336 h of exposure at -20 ℃.


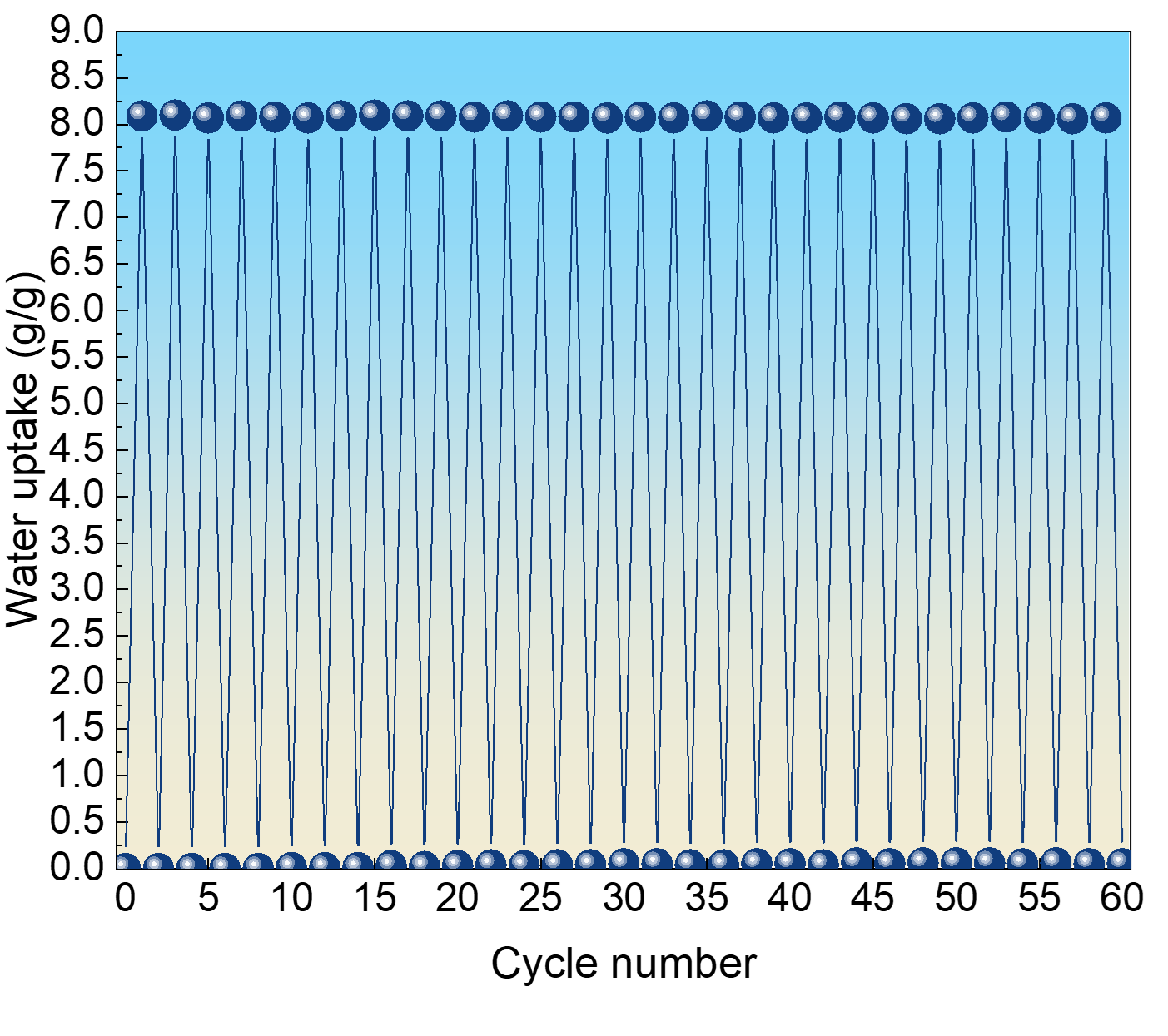


**Figure S7.** Cyclic stability of the BASF after 336 h of exposure at -20 ℃.


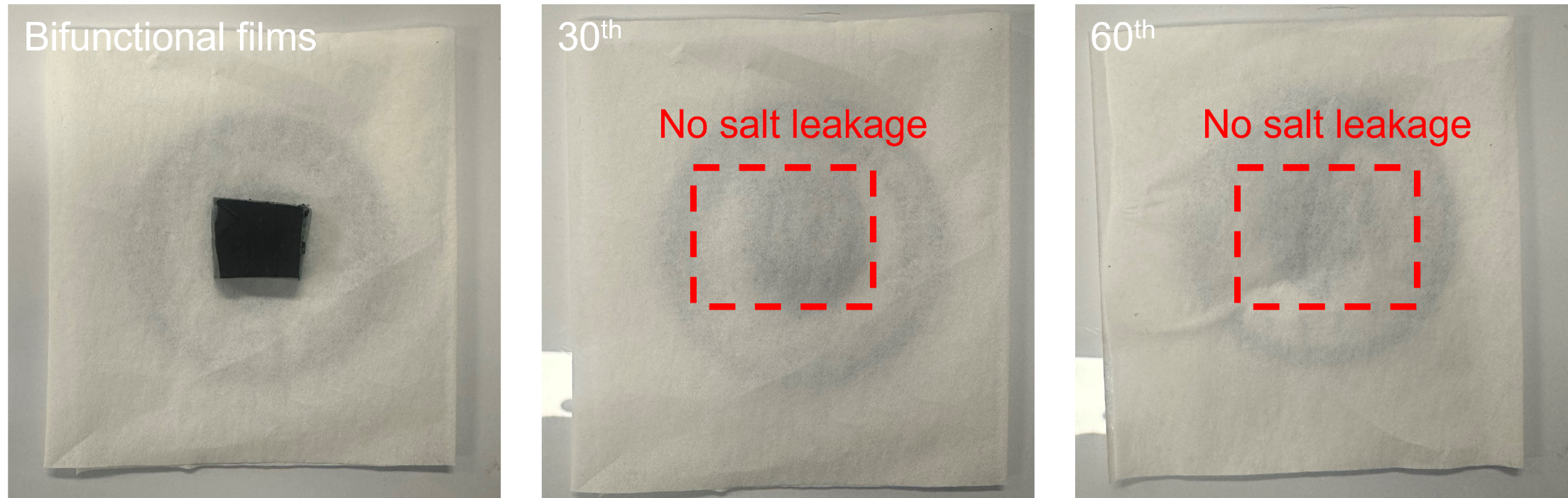


**Figure S8.** BASF exhibits no observable salt leakage after multiple absorption-desorption cycles (adsorption at 20 °C, 90% RH, desorption at 80 °C, 20% RH).


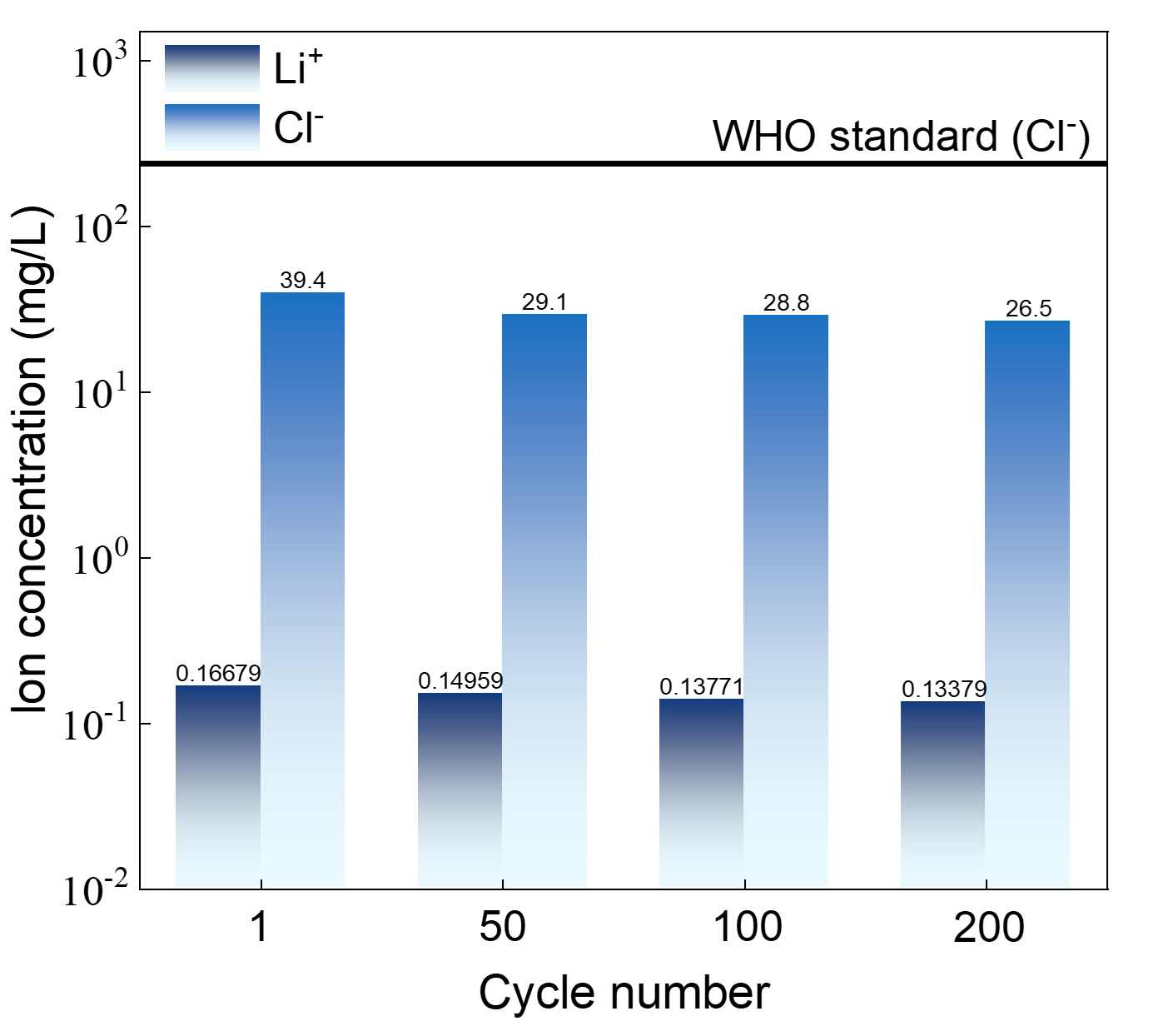


**Figure S9.** Li^+^ and Cl^-^ concentrations in the collected condensate from BASF over repeated atmospheric water-harvesting cycles (adsorption at 20 °C, 90% RH, desorption at 90 °C, 20% RH).

The concentrations of Li^+^ and Cl^-^ in the collected condensate remain low throughout prolonged cycling and show a slight decreasing trend from the 1st to the 200th cycle. Specifically, the Li^+^ concentration decreases from 0.16679 to 0.13379 mg L^-1^, while the Cl^-^ concentration decreases from 39.4 to 26.5 mg L^-1^. No increasing trend is observed with cycle numbers, indicating that LiCl leakage does not accumulate during repeated operation. The slightly higher ion concentration in the first cycle is likely attributed to trace surface residues, whereas the subsequent stable low values confirm the effective confinement of hygroscopic salt within the BASF. These results quantitatively demonstrate the excellent leak-proof stability of the BASF during long-term atmospheric water harvesting.


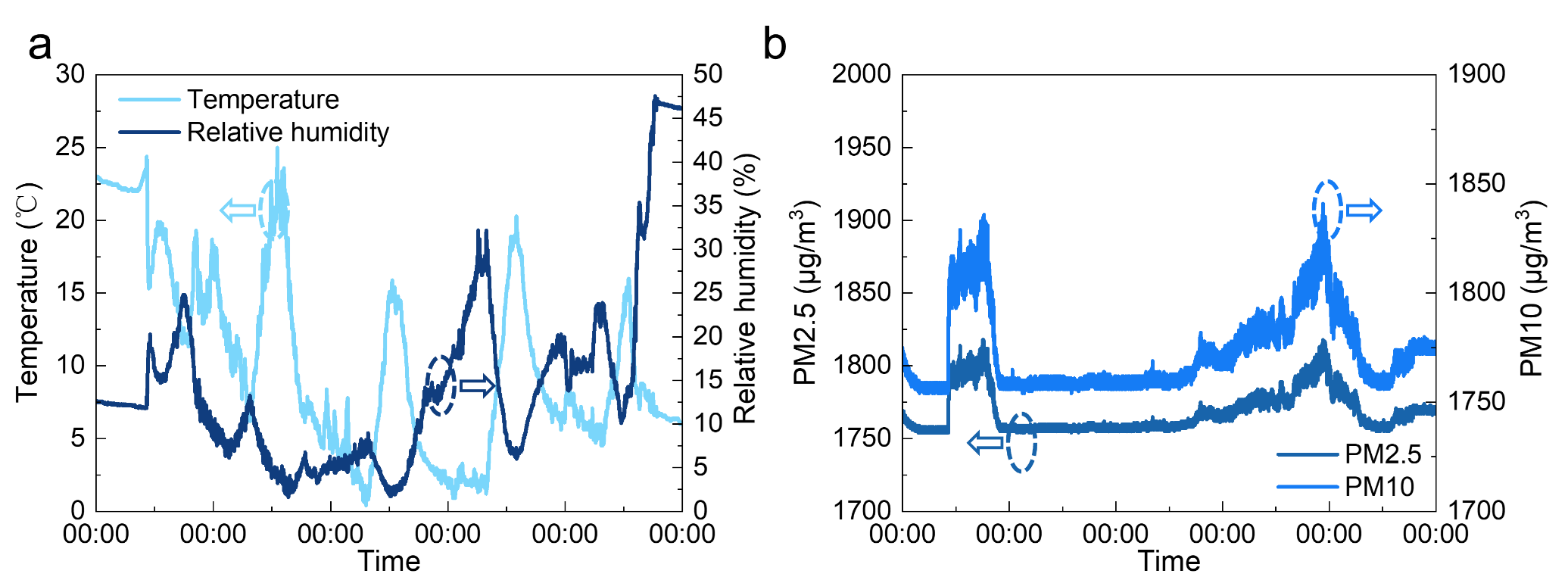


**Fig. S10. Environmental conditions during high-pollution exposure of the BASF. (a)** Variations in ambient temperature and relative humidity. **(b)** Concentration profiles of PM_2.5_ and PM_10_ during the exposure period. (PM > 1500 μg·m^-3^, 5 days).


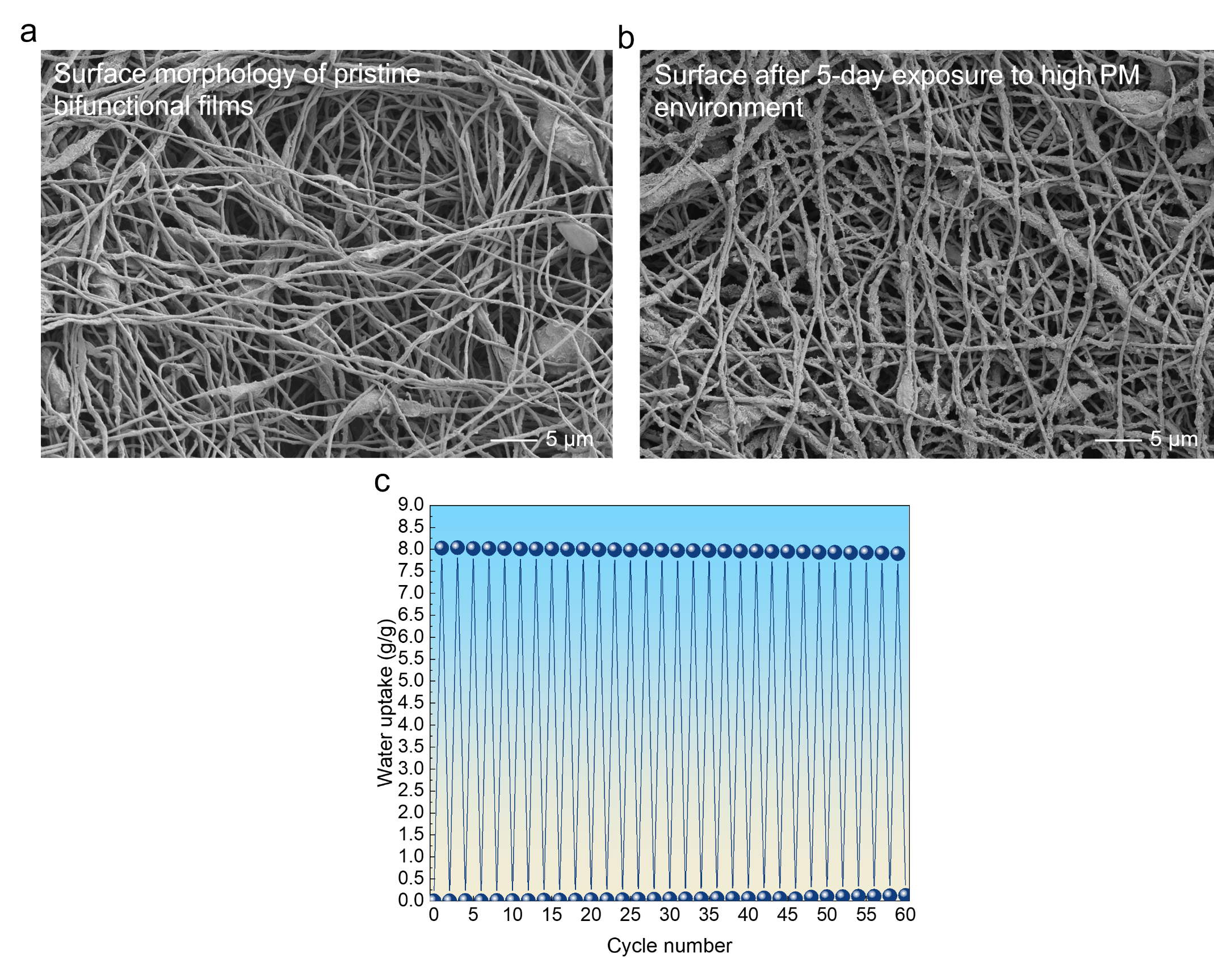


**Fig. S11. SEM images of the BASF before and after prolonged exposure to high concentrations of particulate matter (Particulate Matter (PM) > 1500 μg**·**m^-3^, 5 days), together with the corresponding cyclic stability after PM exposure. (a)** Pristine BASF surface. **(b)** BASF surface after 5-day exposure to a high-PM environment. **(c)** Cyclic stability of the BASF following PM exposure.


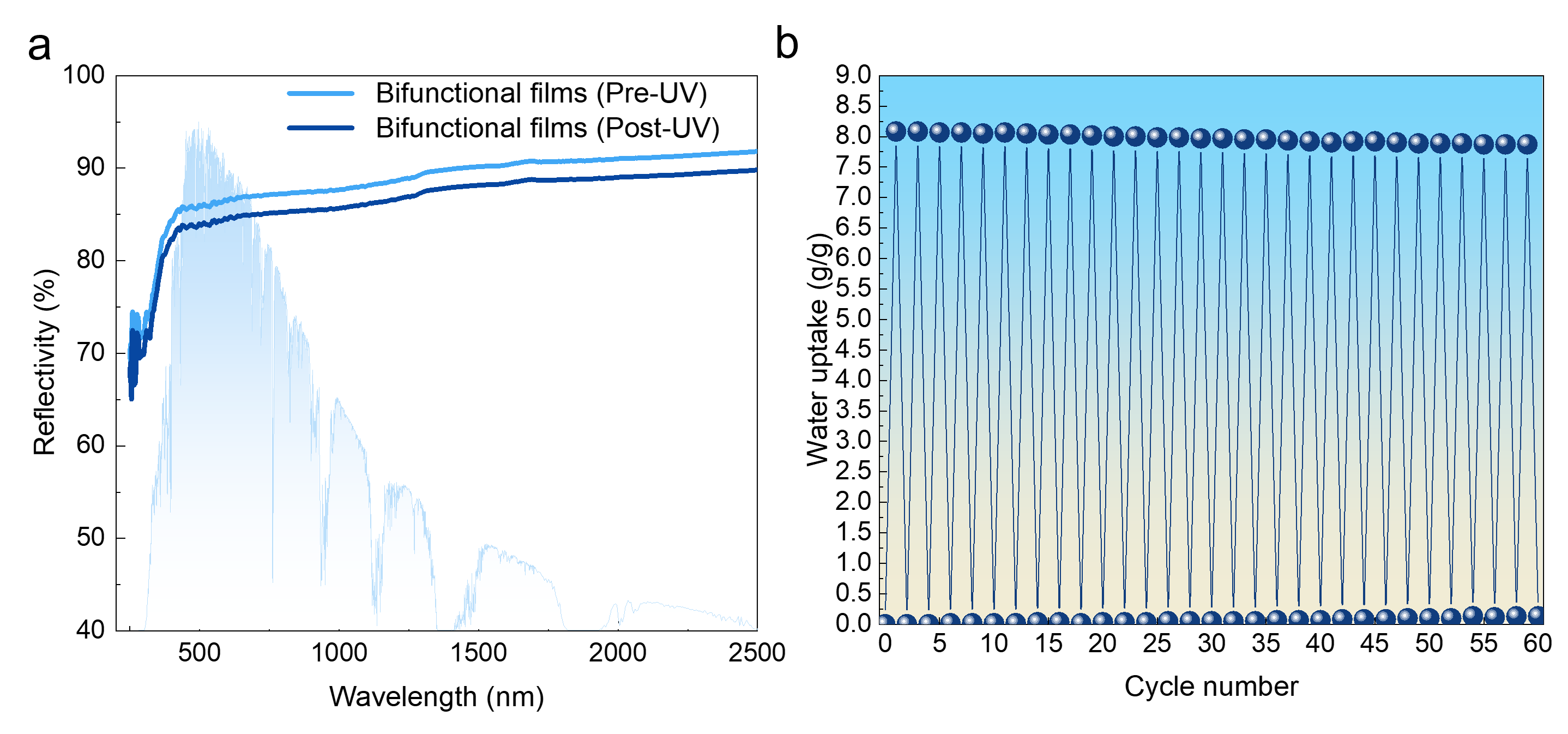


**Figure S12.** **(a)** Comparison of the absorption spectra of the BASF before and after ten years of simulated UV irradiation. **(b)** Cyclic stability of the BASF after UV exposure.


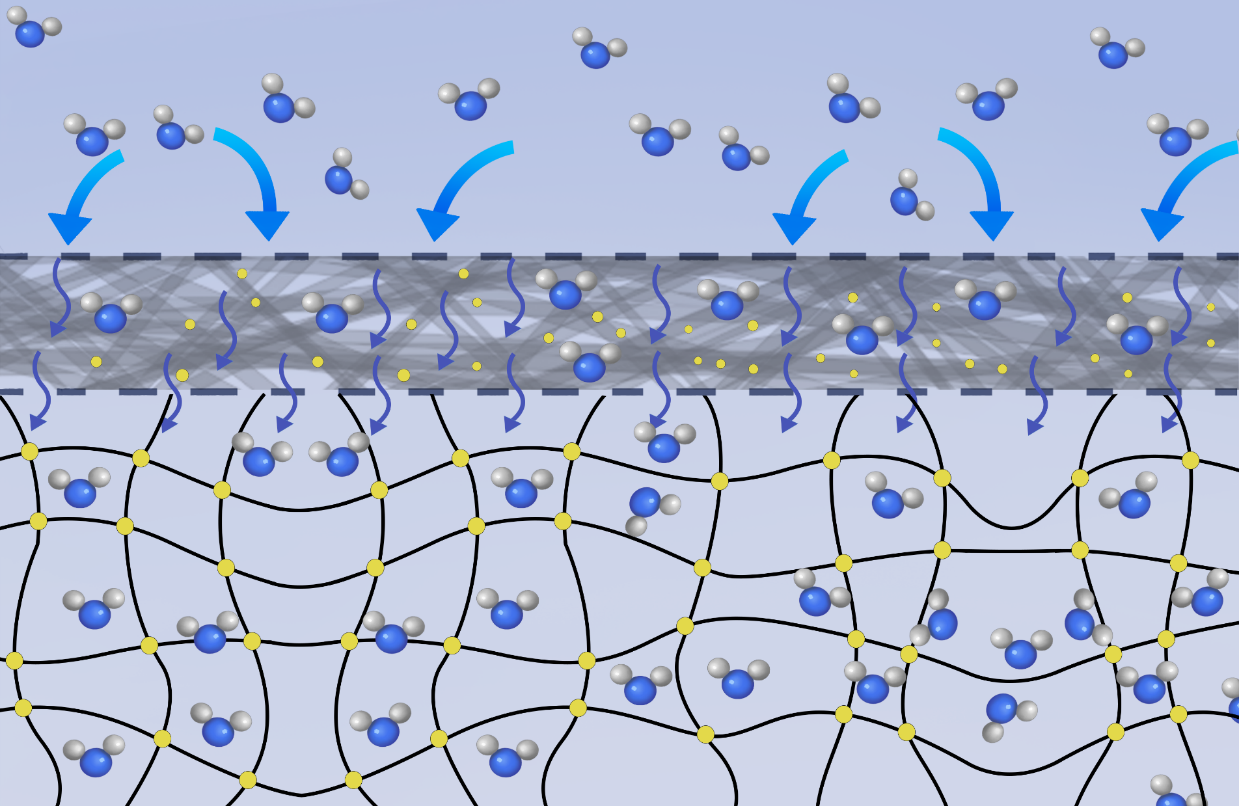


**Figure S13.** A schematic diagram of water molecule adsorption mechanism of the BASF.


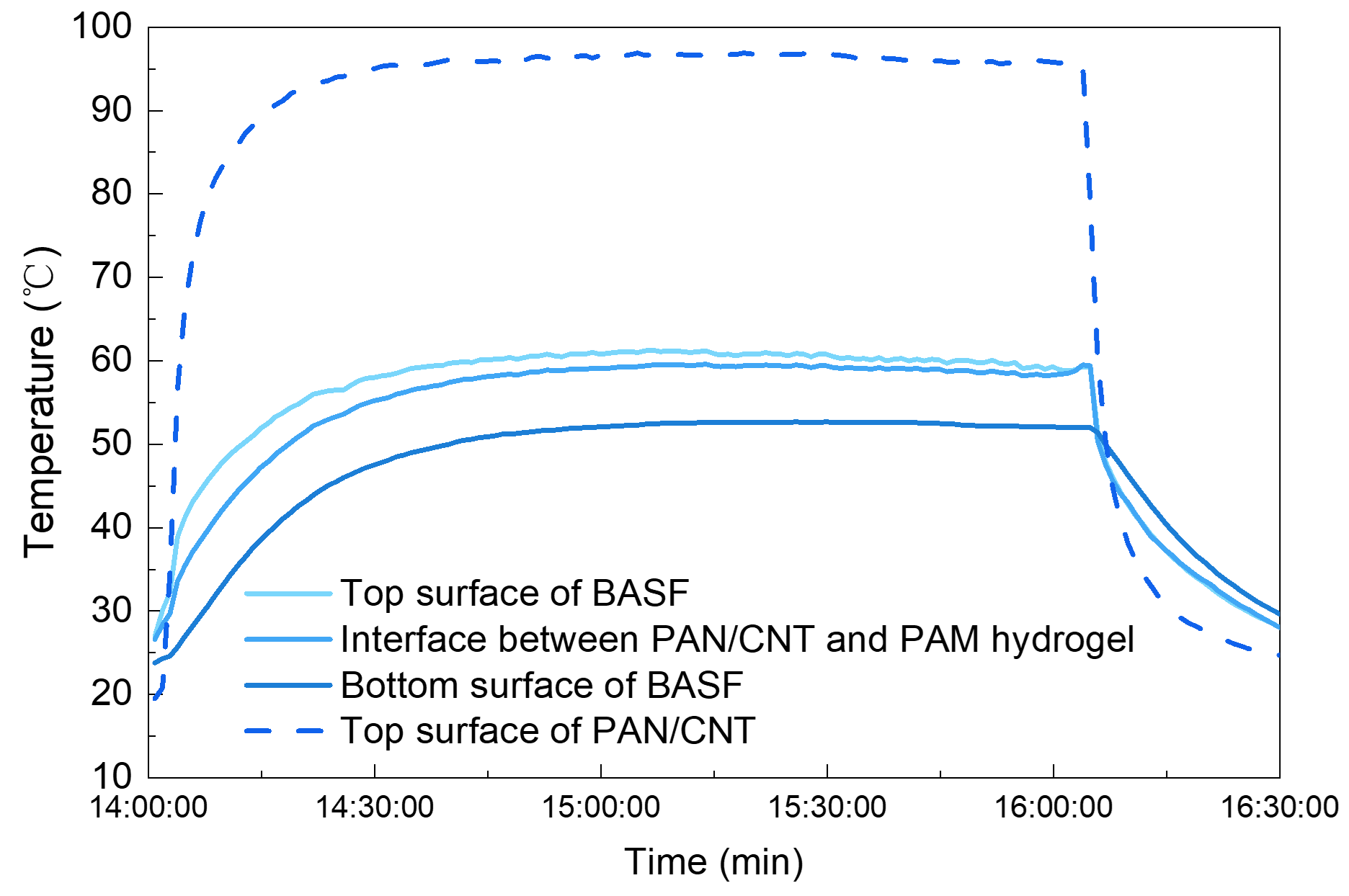


**Fig. S14.** Cross-sectional temperature profiles under simulated solar irradiation.


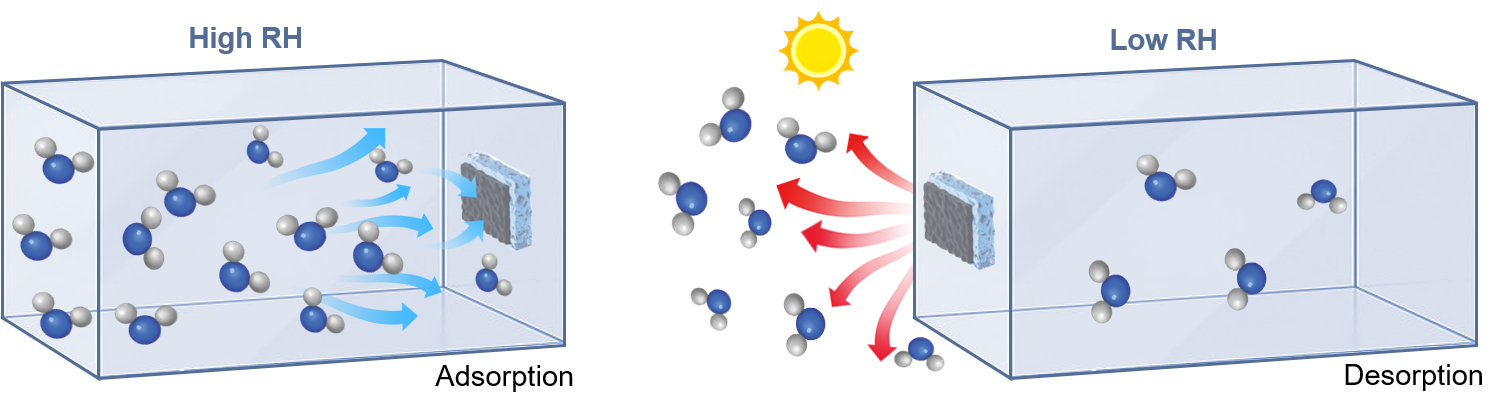


**Figure S15.** Schematic of passive dehumidification in sealed chamber.


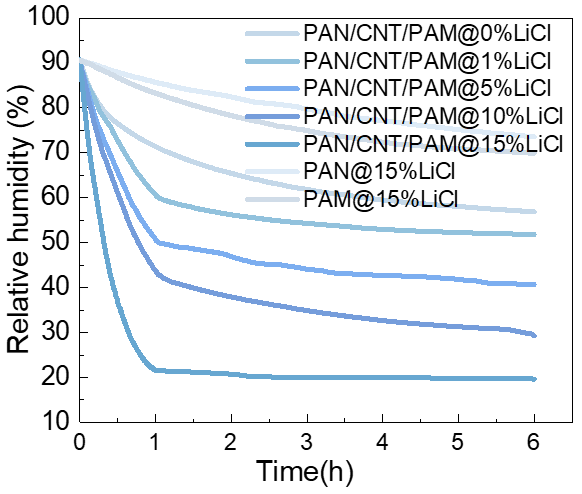


**Figure S16.** Comparative analysis of the dehumidification performance of BASF with varying LiCl concentrations.


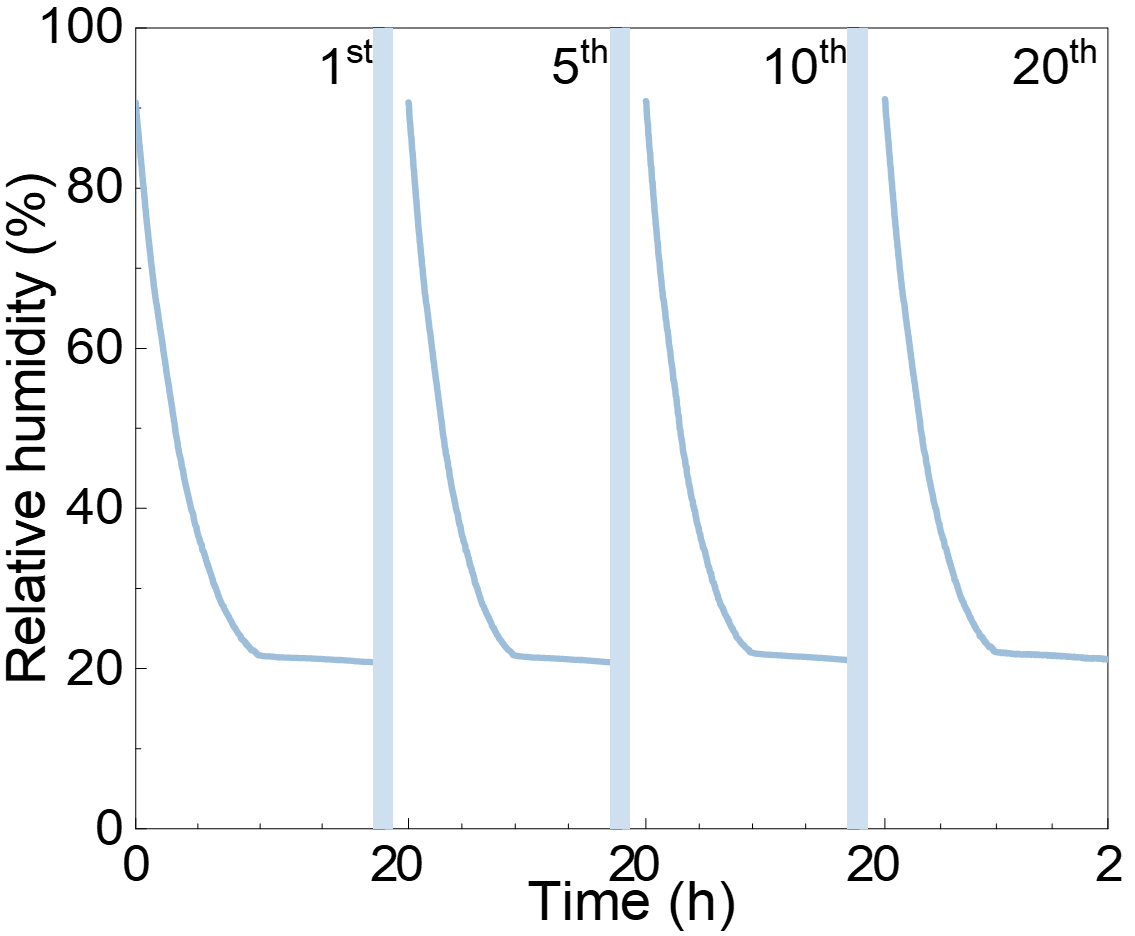


**Figure S17.** Demonstration of stable and reproducible dehumidification performance of the BASF over repeated operational cycles.
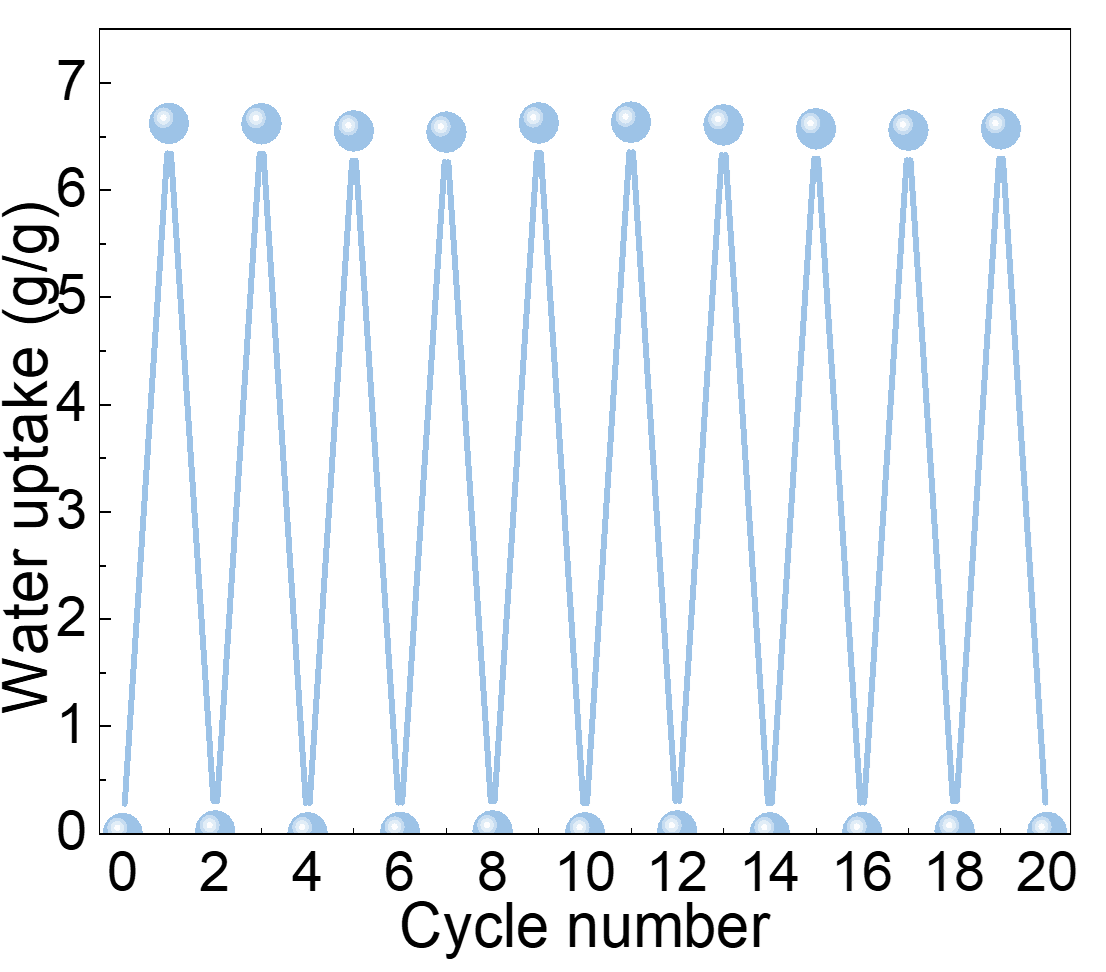


**Figure 18.** Cycling durability of moisture adsorption-desorption at 30 °C and 60% RH, with the desorption conducted at 60 ℃.


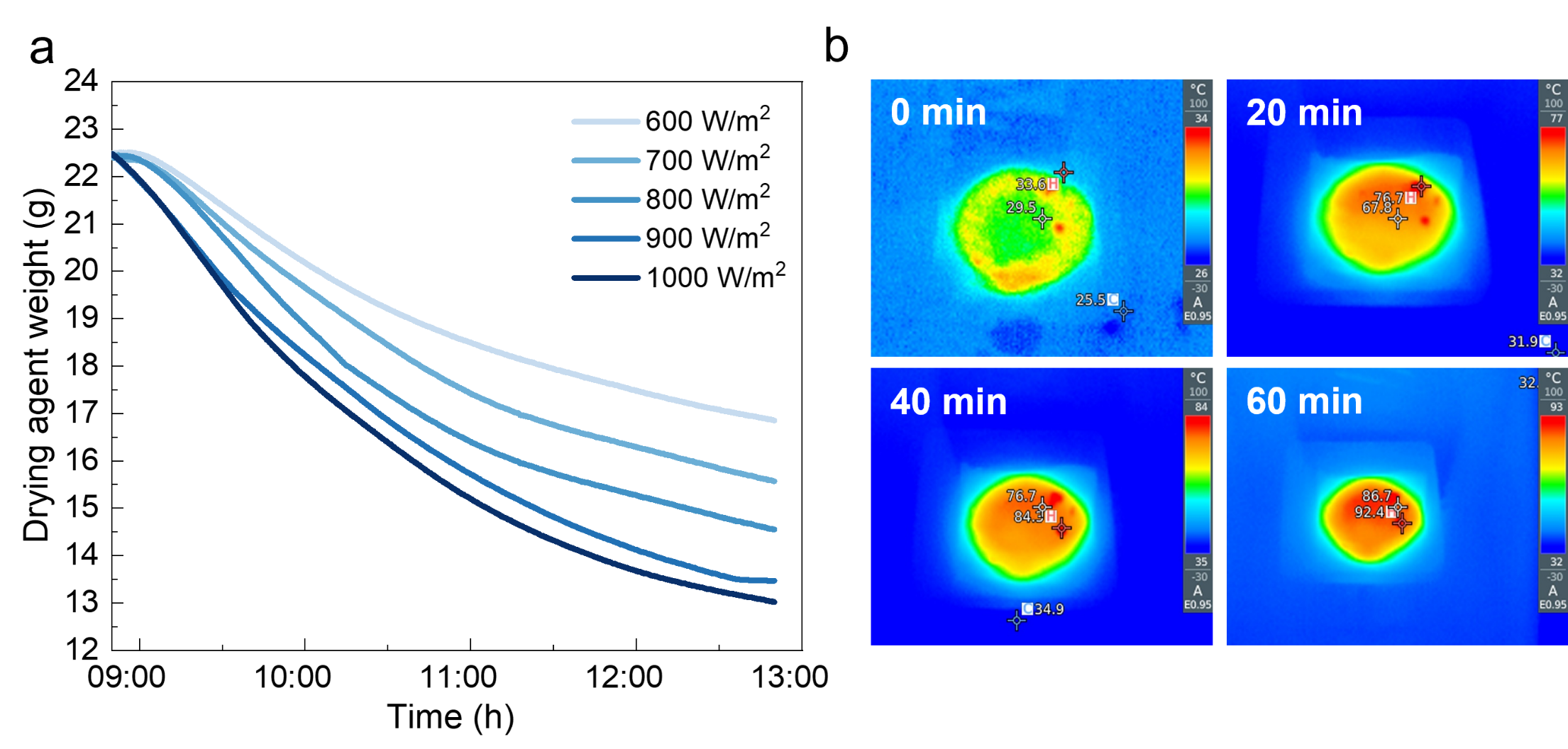


**Figure S19.** **(a)** Desorption performance at different simulated solar intensities. **(b)** Infrared image of surface temperature under the simulated solar irradiation of 1000 W·m^-2^.


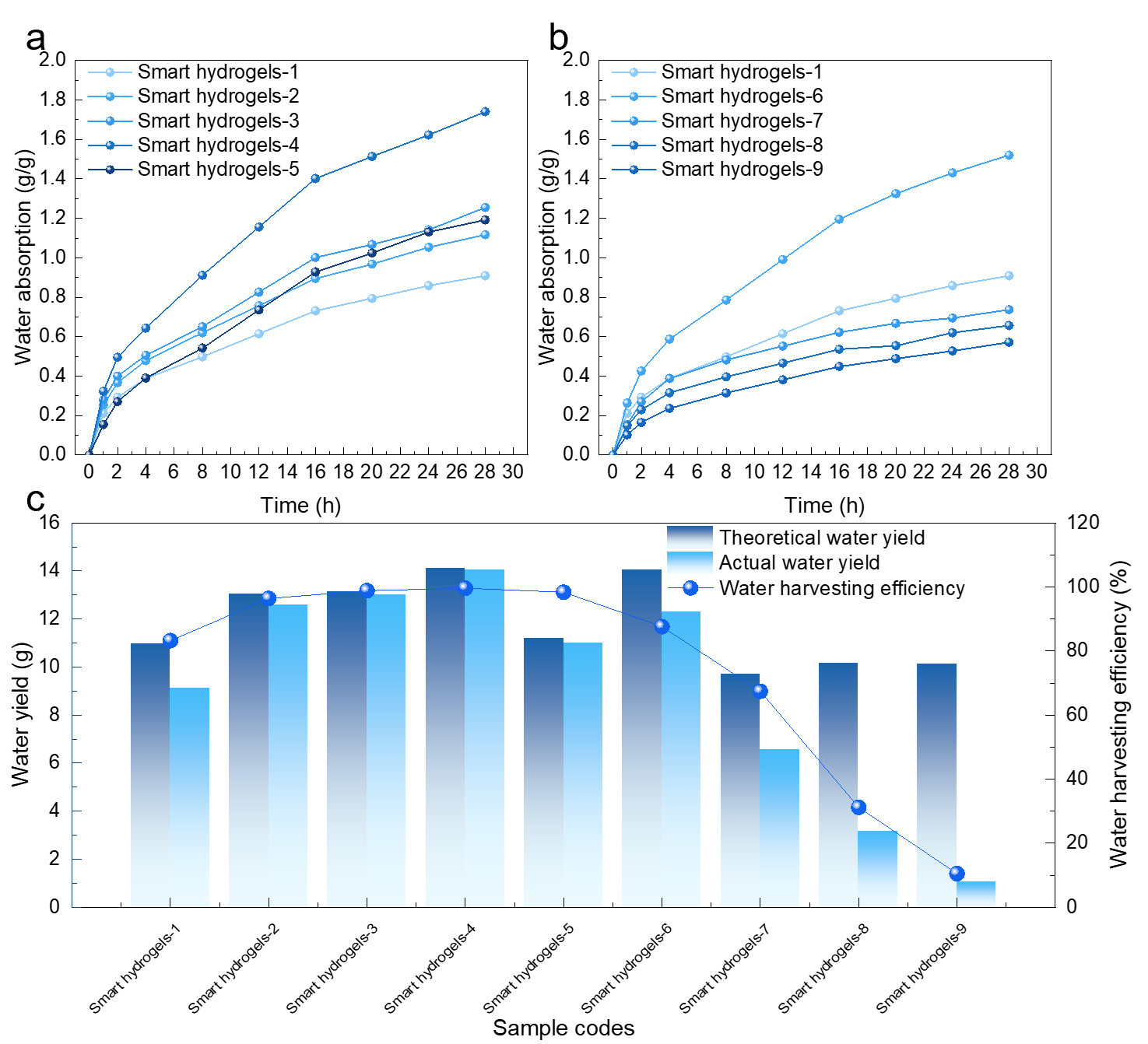


**Figure S20.** **(a)** The effect of different PVA content on the water absorption of smart hydrogels. **(b)** The effect of different AM content on the water absorption of smart hydrogels. **(c)** Water yield of different smart hydrogels.
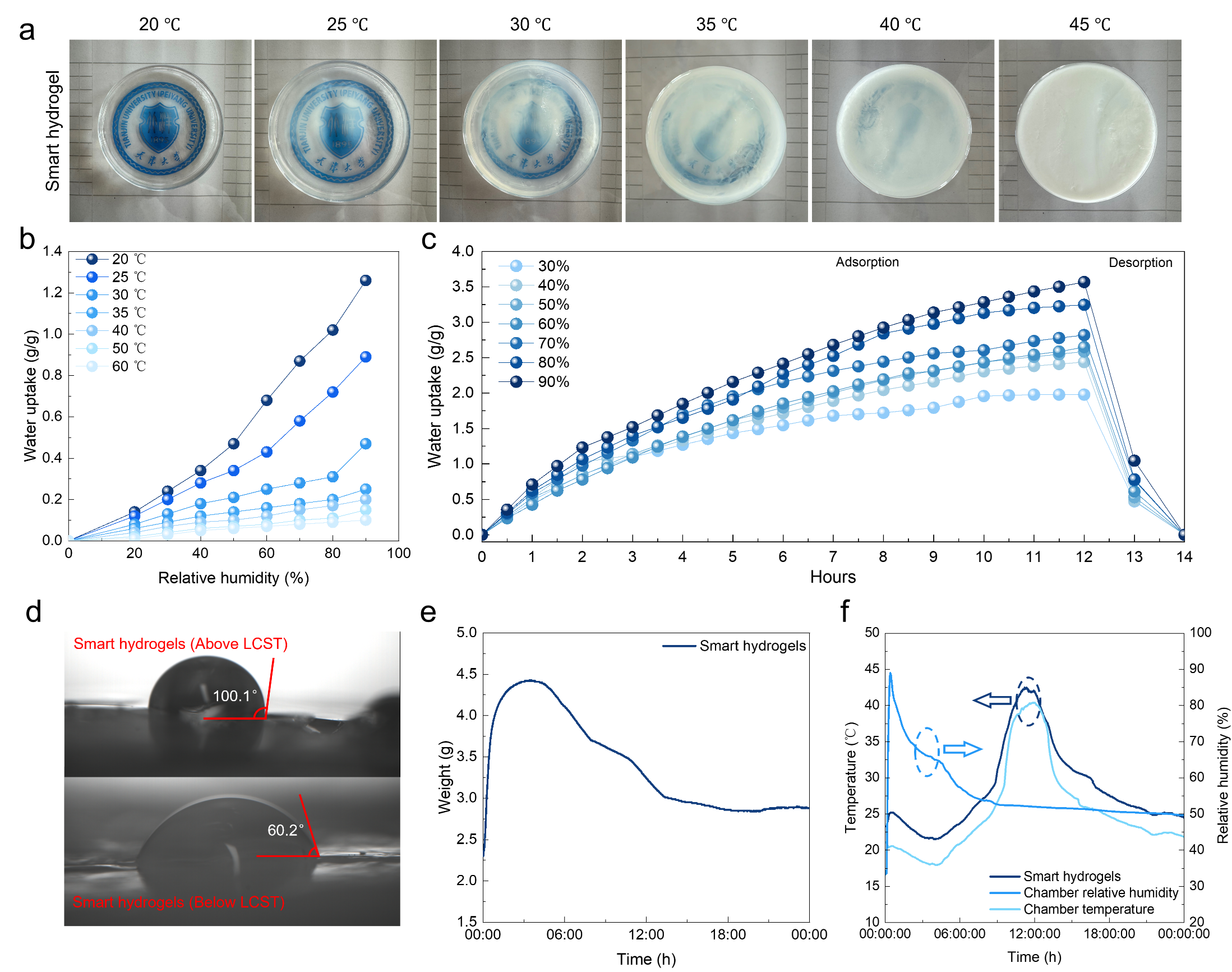


**Figure S21.** **Thermo-responsive hygroscopic behavior and daily indoor humidity-regulation performance of smart hydrogels. (a)** Phase-transition temperature of the smart hydrogels. **(b)** Moisture uptake of salt-free smart hydrogels at different temperatures and relative humidities. **(c)** Moisture adsorption and release behavior of 15 wt% LiCl-loaded smart hydrogels during a 12 h adsorption and 2 h release process. **(d)** Water contact angles before and after the phase transition. **(e)** One-day indoor dehumidification performance of the smart hydrogels. **(f)** One-day temperature profile of the smart hydrogels.

**Figure S21** further confirms the thermo-responsive hygroscopic behavior of the smart hydrogels. The hydrogel shows an evident phase transition with a distinct change in appearance (**Figure S21a**), indicating a temperature-triggered structural transformation. For the salt-free hydrogel, the moisture uptake increases with increasing RH but decreases markedly with increasing temperature (**Figure** **S21b**), demonstrating that water adsorption is favored under low-temperature and high-humidity conditions. After loading 15 wt% LiCl, the smart hydrogel exhibits a clear adsorption-release cycle during the 12 h adsorption and 2 h release process (**Figure S21c**), where substantial moisture is captured during adsorption and rapidly released upon heating. Meanwhile, the water contact angle changes significantly from 60.2° to 100.1° after the phase transition (**Figure S21d**), indicating a pronounced hydrophilic-to-hydrophobic switch. The one-day indoor humidity-regulation and temperature profiles (**Figures S21e and f**) further suggest that the hydrogel can respond dynamically to indoor environmental fluctuations, thereby supporting its potential for autonomous humidity management and water-release applications.


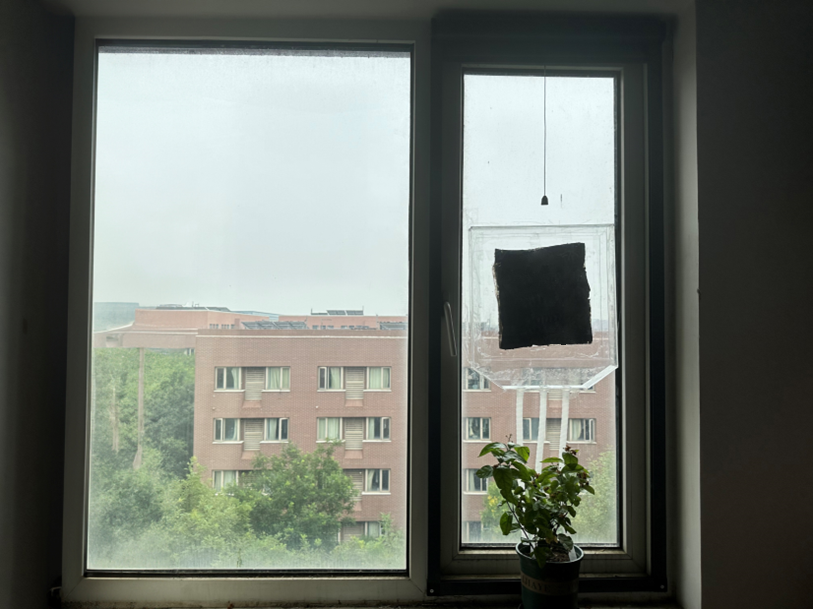


**Figure S22.** The dehumidification experiment was performed in a sealed room using BASF.


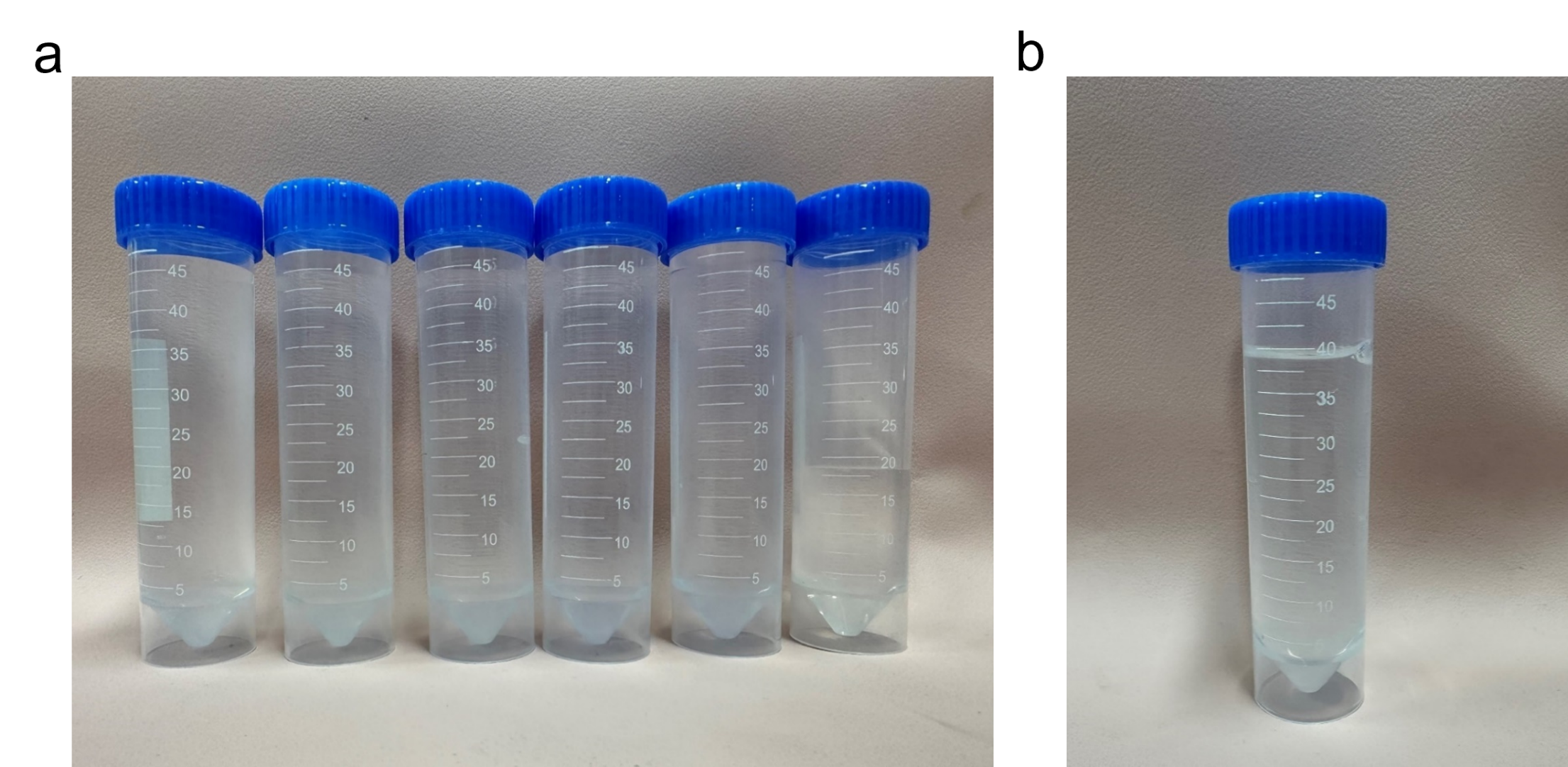


**Figure S23.** **(a)** The daily water production of BASF in indoor environments. **(b)** The daily water production of smart hydrogel in indoor environments.


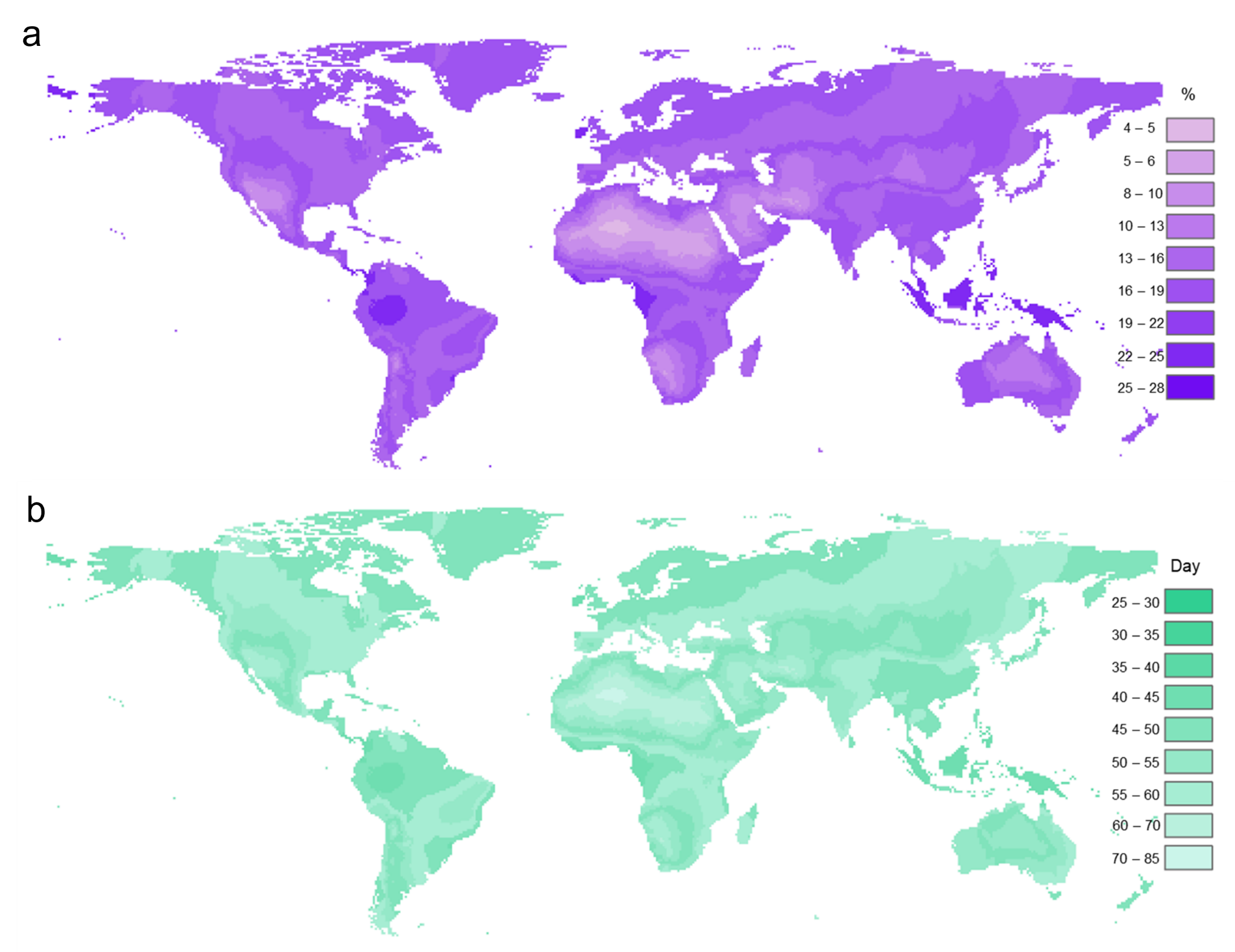


**Figure S24.** **(a)** Annual energy saving rate from humidity management for 50 m^2^ buildings. **(b)** Payback time from humidity management for 50 m^2^ buildings.


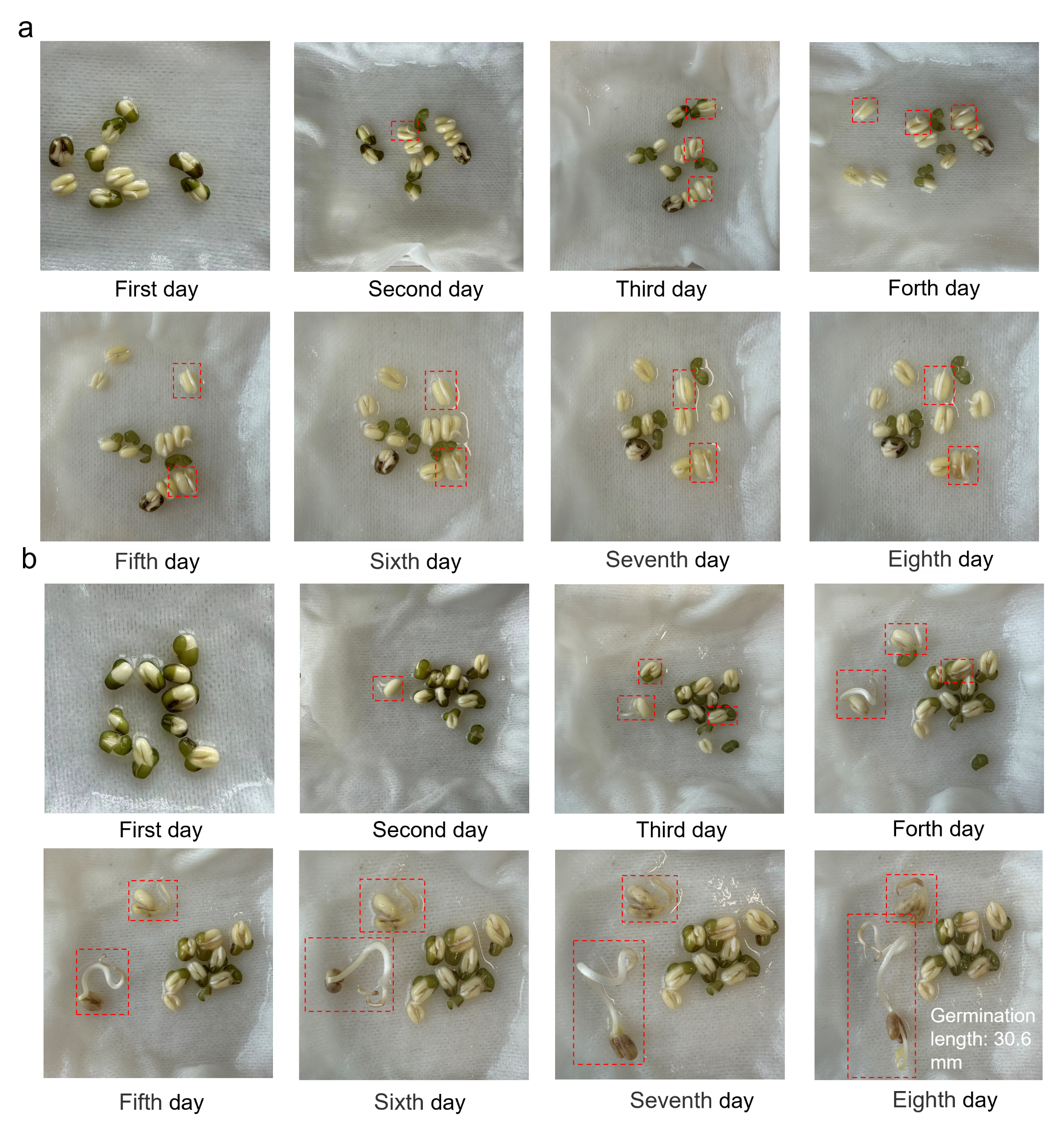


**Figure S25.** **(a)** Cultivation using regular tap water. **(b)** Cultivation using atmospheric water.


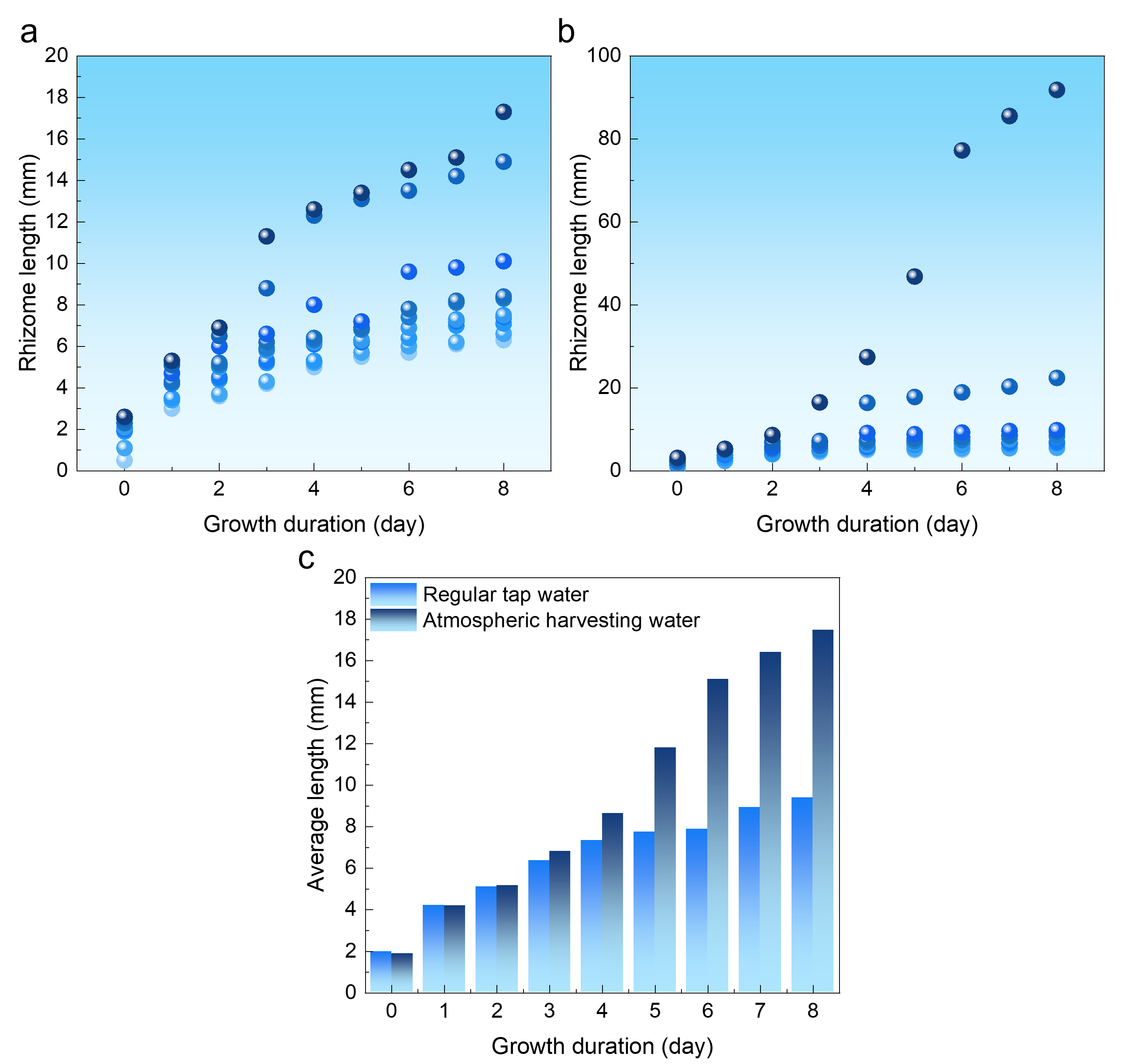


**Figure S26.** **(a)** Results of 8-day cultivation using tap water. **(b)** Results of 8-day cultivation using atmospheric harvesting water. **(c)** Comparative cultivation results in using different water sources.

# References

1. M. Wu, Y. Zhou, S. Aleid, X. Tang, Y. Zhao, R. Li, P. Wang, *ACS Sustainable. Chem. Eng*, **2024**, *12*, 1255-1264.
2. C. Liu, L. Xu, Z. Wang, S. Han, M. Fu, B. Yuan, *Langmuir*, **2024**, *40*, 14413-14425.
3. C. Fu, D. Zhan, G. Tian, A. Yu, L. Yao, Z. Guo, *ACS Appl. Mater. Interfaces*, **2024**, *16*, 35740-35751.
4. S. Guan, C. Xu, X. Dong, M. Qi, *J. Mater. Chem. A*, **2023**, *11*, 15404-15415.
5. J. Wang, C. Deng, G. Zhang, W. Ying, C. Li, S. Wang, Y. Liu, R. Wang and H. Zhang, *Cell. Rep. Phy. Sci*, **2022**, *3*, 100954.
6. P. Schweng, C. Li, P. Gugenberger, F. Kleitz and R. T. Woodward, *ChemSusChem*, **2024**, *17*, e202301906.
7. W. Guan, Y. Zhao, C. Lei, Y. Wang, K. Wu and G. Yu, *Adv. Mater*, **2025**, 2420319.
8. Y. Li, J. Deng and H. Li, *Appl. Therm. Eng*, **2024**, *247*, 123045.
9. M. Solovyeva, I. Krivosheeva, L. Gordeeva and Y. Aristov, *Energies*, **2021**, *14*, 3586.
10. Z. Zhou, Y. Zhang, W. Liu, C. Gui, L. Huang, H. Huang, K. Fan, Y. Huang, Y. Gong, A. Chen, P. Liu and H. Jiang, *Desalination*, **2024**, *583*, 117685.
11. A. Feng, Y. Shi, C. Onggowarsito, X. S. Zhang, S. Mao, M. A. H. Johir, Q. Fu and L. D. Nghiem, *ChemSusChem*, **2024**, *17*, e202301905.
12. M. Dai, F. Zhao, J. Fan, Q. Li, Y. Yang, Z. S. Fan, H. Yu, S. Liu, J. Li, W. Chen and G. Yu, *Adv. Mater*, **2022**, *34*, 2200865.
13. Y. Hu, Y. Wang, Z. Fang, X. Wan, M. Dong, Z. Ye and X. Peng, *J. Mater. Chem. A*, **2022**, *10*, 15116-15126.
14. F. Luo, X. Liang, W. Chen, S. Wang, X. Gao, Z. Zhang and Y. Fang, *Chem. Eng, J*, **2022**, *450*, 138241.
15. Y. Li, H. Chen, R. Deng, M. Wu, H. Yang and S. B. Darling, *ACS Appl. Mater. Interfaces*, **2021**, *13*, 33713-33721.
16. C. Cai, Y. Chen, F. Cheng, Z. Wei, W. Zhou and Y. Fu, *ACS Nano*, **2024**, *18*, 4376-4387.
17. X. Zhang, J. Yang, H. Qu, Z. Yu, D. K. Nandakumar, Y. Zhang and S. C. Tan, *Adv. Sci*, **2021**, *8*, 2003939.
18. K. Zhang, X. Lei, C. Mo, J. Huang, M. Wang, E. Kang and L. Xu, *Adv. Sci*, **2023**, *10*, 2206925.
19. Y. Nong, B. Fan, X. Bao, B. Xu, M. Zhou, Y. Yu, Q. Wang and P. Wang, *Mater. Today. Commun*, **2022**, *32*, 103984.
20. J. Fu, Y. Liu, L. Chen, W. Han, X. Liu, J. Shao, X. Yan and Z. Gu, *Small*, **2023**, *19*, 2303897.
21. Y. Lin, K. Shao, S. Li, N. Li, S. Wang, X. Wu, C. Guo, L. Yu, P. Murto and X. Xu, *ACS Appl. Mater. Interfaces*, **2023**, *15*, 10084-10097.
22. H. Wu, Y. Xiong, D. Yu, P. Yang, H. Shi, L. Huang, Y. Wu, M. Xi, P. Xiao and L. Yang, *Nanoscale*, **2022**, *14*, 18022-18032.
23. M. Wang, T. Sun, D. Wan, M. Dai, S. Ling, J. Wang, Y. Liu, Y. Fang, S. Xu, J. Yeo, H. Yu, S. Liu, Q. Wang, J. Li, Y. Yang, Z. Fan and W. Chen, *Nano. Energy*, **2021**, *80*, 105569.
24. Z. Zhou, G. Wang, X. Pei and L. Zhou, *Chem. Eng. J*, **2023**, *474*, 145605.
25. J. Xu, T. Li, J. Chao, S. Wu, T. Yan, W. Li, B. Cao and R. Wang, *Angew. Chem. Int. Ed*, **2020**, *59*, 5202-5210.
26. C. Liu, L. Xu, Z. Wang, S. Han, M. Fu and B. Yuan, *Langmuir*, **2024**, *40*, 14413-14425.
27. F. Deng, C. Wang, C. Xiang and R. Wang, *Nano. Energy*, **2021**, *90*, 106642.
28. M. Wu, Y. Zhou, S. Aleid, X. Tang, Y. Zhao, R. Li, P. Wang, *ACS Sustainable. Chem. Eng*, **2024**, *12*, 1255-1264.
29. Y. Hu, Z. Fang, X. Wan, X. Ma, Y. Wang, M. Dong, Z. Ye and X. Peng, *ACS Sustainable. Chem. Eng*, **2022**, *10*, 6446-6455.
30. Q. Liu, C. Qin, Q. Zhu, W. Wu and X. Wang, *Int. Commun. Heat. Mass*, **2024**, *155*, 107579.
31. Y. Hu, Y. Wang, Z. Fang, B. Yao, Z. Ye and X. Peng, *ACS Appl. Mater. Interfaces*, **2023**, *15*, 44942-44952.
32. Z. Zhang, Y. Wang, Z. Li, H. Fu, J. Huang, Z. Xu, Y. Lai, X. Qian and S. Zhang, *ACS Appl. Mater. Interfaces*, **2022**, *14*, 55295-55306.
33. P. Zhu, Z. Yu, H. Sun, D. Zheng, Y. Zheng, Y. Qian, Y. Wei, J. Lee, S. Srebnik, W. Chen, G. Chen and F. Jiang, *Adv. Mater*, **2023**, *36*, 2306653.
34. X. Han, L. Zhong, L. Zhang, L. Zhu, M. Zhou, S. Wang, D. Yu, H. Chen, Y. Hou and Y. Zheng, *Small*, **2023**, *19*, 2303358.
35. D. Chakraborty, A. Yurdusen, G. Mouchaham, F. Nouar and C. Serre, *Adv. Funct. Mater*, **2023**, *34*, 2309089.
36. H. Shan, C. Li, Z. Chen, W. Ying, P. Poredos, Z. Ye, Q. Pan, J. Wang and R. Wang, *Nat. Commun*, **2022**, *13*, 5406.
37. K. Matsumoto, N. Sakikawa and T. Miyata, *Nat. Commun*, **2018**, *9*, 2315.
38. G. Yilmaz, F. Meng, W. Lu, J. Abed, C. K. N. Peh, M. Gao, E. H. Sargent and G. W. Ho, *Sci. Adv*, **2020**, *6*, 42.
39. H. Lu, W. Shi, J. H. Zhang, A. C. Chen, W. Guan, C. Lei, J. R. Greer, S. V. Boriskina and G. Yu, *Adv. Mater*, **2022**, *34*, 2205344.
40. J. Zhang, R. Bhat and K. D. Jandt, *Acta. Biomater*, **2009**, *5*, 488-497.
41. Y. Zhang, L. Wu, X. Wang, J. Yu and B Ding, *Nat. Commun*, **2020**, *11*, 3302.
42. M. Wang, T. Sun, D. Wan, M. Dai, S. Ling, J. Wng, Y. Liu, Y. Fang, S. Xu, J. Yeo, H. Yu, S. Liu, Q. Wang, J. Li, Y. Yang, Z. Fan and W. Chen, *Nano. Energy*, **2021**, *80*, 105569.
43. W. Guan, Y. Zhao, C. Lei and G. Yu, *P. Natl. Acad. Sci*, **2023**, *120*, e2308969120.
44. Y. Hu, Y. Wang, Z. Fang, B. Yao, Z. Ye and X. Peng, *ACS Appl. Mater. Interfaces*, **2023**, *15*, 44942-44952.
45. P. Zhu, Z. Yu, H. Sun, D. Zheng, Y. Zhen, Y. Qian, Y. Wei, J. Lee, S. Srebnik, W. Chen, G. Chen and F. Jiang, *Adv. Mater*, **2023**, *36*, 2306653.
